# Phenazines are involved in the antagonism of a novel subspecies of *Pseudomonas chlororaphis* strain S1Bt23 against *Pythium ultimum*

**DOI:** 10.1038/s41598-024-71418-y

**Published:** 2024-09-03

**Authors:** Sylvia I. Chi, Mercy Akuma, Renlin Xu, Véronique Plante, Mehri Hadinezhad, James T. Tambong

**Affiliations:** 1 Ottawa Research and Development Centre, Agriculture and Agri-Food Canada, Ottawa, ON K1A 0C6 Canada; 2 Canadian Blood Service, Ottawa, ON K1G 4J5 Canada; 3 University of Ottawa, Ottawa, ON K1N 6N5 Canada; 4 Department of Plant Science, University of Manitoba, Winnipeg, MB R3T 2N2 Canada

**Keywords:** Biological control, Phenazine, Phenazine-1-carboxylic acid, *Pseudomonas chlororaphis*, CRISPR/cas9, *Pseudomonas chlororaphis* subsp. *phenazini* subsp. nov., *Pythium ultimum*, Secondary metabolites, Microbiology, Applied microbiology, Bacteria, Bacteriology, Pathogens, Comparative genomic hybridization, Comparative genomics

## Abstract

Long-term use of chemical fungicides to control plant diseases caused by fungi and oomycetes has led to pathogen resistance and negative impacts on public health and environment. There is a global search for eco-friendly methods and antagonistic bacteria are emerging as alternatives. We isolated a potent antagonistic bacterial strain (S1Bt23) from woodland soil in Québec, Canada. Taxonomic characterization by 16S rRNA, multi-locus sequence analysis, pairwise whole-genome comparisons, phylogenomics and phenotypic data identified strain S1Bt23 as a novel subspecies within *Pseudomonas chlororaphis*. In dual culture studies, strain S1Bt23 exhibited potent mycelial growth inhibition (60.2–66.7%) against *Pythium ultimum.* Furthermore, strain S1Bt23 was able to significantly bioprotect potato tuber slices from the development of necrosis inducible by *P. ultimum*. Annotations of the whole genome sequence of S1Bt23 revealed the presence of an arsenal of secondary metabolites including the complete phenazine biosynthetic cluster (*phz*ABCDEFG). Thin-layer (TLC) and high-performance liquid (HPLC) chromatographic analyses of S1Bt23 extracts confirmed the production of phenazines, potent antifungal compounds. CRISPR/Cas9-mediated deletion of *phz*B (S1Bt23Δ*phz*B) or *phz*F (S1Bt23Δ*phz*F) gene abrogated phenazine production based on TLC and HPLC analyses. Also, S1Bt23Δ*phz*B and S1Bt23Δ*phz*F mutants lost antagonistic activity and bioprotection ability of potato tubers against *P. ultimum*. This demonstrated that phenazines are involved in the antagonistic activity of S1Bt23 against *P. ultimum*. Finally, based on genotypic and phenotypic data, we taxonomically conclude that S1Bt23 represents a novel subspecies for which the name *Pseudomonas chlororaphis* subsp. *phenazini* is proposed.

## Introduction

Field and greenhouse crops incur significant yield losses annually due to biotic stressors such as fungi, oomycetes, bacteria and viruses^1^. Fungal and oomycetes pathogens are often the most prevalent culprits of these yield losses by infecting seeds and plants throughout the growth cycle leading to typical symptoms, such as chlorosis, leaf spots, necrosis, wilting and growth inhibition^2^. Fungal and oomycetous phytopathogens such as *Alternaria solani*, *Rhizoctonia solani* and *Pythium* species cause plant diseases worldwide^3,4^. Several *Pythium* species, including *P. aphanidermatum*, *P. irregulare* and *P. ultimum*, infect greenhouse and field cucumber, pepper, tomato and cannabis plants^5–7^. *Pythium ultimum*, re-classified as *Globisporangium ultimum*^8^, is a major pathogen of hundreds of horticultural and agricultural crops including wheat, soybean and potato^9,10^. Chemical pesticides are routinely used to control these pathogens^11^. This long-term and sustained use of these synthetic chemical pesticides have resulted in pathogen resistance, elimination of beneficial microorganisms^12^, public health and environmental issues and destabilization of some aquatic and terrestrial ecosystems^13,14^. Due to these negative impacts, there is global consensus for the search of eco-friendly control methods and bacteria-based products are emerging as valuable alternatives. Several in vitro and in vivo studies have reported inhibition and effective reduction of the incidences of important phytopathogens by the antagonistic action of specific bacterial species within the genus *Pseudomonas*^15–19^.

The genus *Pseudomonas* (phylum, *Pseudomondota*; class *Gammaproteobacteria*) consists of 351 validly published species and 10 subspecies (as of August 15, 2024) of Gram-negative, aerobic, facultatively anaerobic rod-shaped bacteria^20,21^. These bacteria are versatile and can colonize different ecological niches such as rhizosphere, soils, water and organisms (plants, animals and humans)^11,21,22^. Most species of fluorescent pseudomonads play significant ecological roles in agricultural and environmental sustainability such as xenobiotic-degraders, biological antagonists and plant growth promoters^1,21,22^. These properties are key to effective biological control agents, thus, making *Pseudomonas* strains the focus of several biopesticide research efforts^21,23,24^. Most promising *Pseudomonas* biocontrol species are effective against many fungal and oomycetes pathogens of important crops by producing antifungal or antimicrobial metabolites such as phenazine-1-carboxylic acid (PCA), 2-hydroxyphenazine, 2,4-diacetylphloroglucinol (2,4-DAPG), pyoluteorins and pyrrolnitrins^25–27^.

In 2015, we initiated a study to investigate the diversity of cultivable bacteria communities associated with Canadian woodlands in Aylmer, Québec, Canada, which resulted to the isolation of 161 strains^28^. The majority (64%) of the isolates were identified as *Pseudomonas* spp. based on 16S rRNA gene sequences. The 16S rRNA BLAST results of one isolate (S1Bt23) hit the type strain of *Pseudomonas chlororaphis* ATCC 9446^T^ as the best and closest GenBank entry with 99.9% sequence similarity. 16S rRNA sequence analysis coupled with colony pigmentation and other phenotypic data provided an indication that strain S1Bt23 might be of interest as *P. chlororaphis* strains often possess an arsenal of biologically active secondary metabolites or antimicrobial compounds that can be potent growth inhibitors of fungi and oomycetes^27,29^. This prompted further investigation of strain S1Bt23. In addition, Loper et al.^30^ and Biessy et al.^31^ studied the phytobeneficial traits of phenazine-producing *Pseudomonas* spp. based on whole-genome data which underscored their genetic diversity.

The taxonomy of members of *P. chlororaphis* has undergone significant revisions over the years. In the Approved List of bacterial names^32^, *Pseudomonas aureofaciens, Pseudomonas aurantiaca* and *Pseudomonas chlororaphis* were classified as separate species. Johnson and Palleroni^33^, using phenotypic traits and DNA relatedness, proposed that *P. aureofaciens* is a later heterotypic synonym of *P. chlororaphis* while Palleroni (2005) classified them as two distinct subspecies. Peix et al.^34^ reclassified *P. aureofaciens*, *P. aurantiaca* and *P. chlororaphis* into three novel subspecies while Burr et al.^35^ validly published *P. chlororaphis* subsp. *piscium* on the basis of wet laboratory DNA–DNA hybridization (wDDH) and phenotypic data. While the wDDH technique was the gold standard in species/subspecies delineation, it had inherent limitations of poor reproducibility. Current bacterial classification based on whole-genome derived parameters, such as digital DNA–DNA hybridization (dDDH), is more reliable and reproducible, and required to provide a more stable taxonomy to this important biological control bacterial species.

The objectives of this study were to (i) investigate the taxonomic position of strain S1Bt23 using multilocus sequence analysis, 81 up-to-date bacterial core genes version 2 (ubcg2^36^) and phylogenomics as well as genome-based DNA–DNA hybridization (DDH) and average nucleotide identity (ANI); (ii) comprehensively mine the whole genome sequence of strain S1Bt23 for secondary metabolite biosynthetic clusters and other virulence factors; and (iii) assess whether strain S1Bt23 can biologically control *Pythium ultimum,* the Pythium leak disease pathogen of potato tubers, in vitro and *in planta* and determine the potential metabolites involved in its antagonistic activity. Genome-based DNA–DNA hybridization values, 16S rRNA-*gyr*B-*rpo*B-*rpo*D concatenated phylogeny, phylogenomics and ubcg2 analyses performed in this study suggest that strain S1Bt23 is valid member of *Pseudomonas chlororaphis* but is unique and a potential novel subspecies. Whole-genome sequence mining using antiSMASH identified 16 secondary metabolite producing clusters consisting of non-ribosomal peptide synthetases (nrps), beta-lactone, siderophore, arylpolyene, dipeptide N-acetylglutaminylglutamine amide (NAGGN), homoserine lactone (hserlactone), pyrrolnitrin and phenazines. TLC and HPLC analyses revealed positive biosynthesis of phenazines by strain S1Bt23. We showed that strain S1Bt23 inhibits the growth *P. ultimum *in vitro and, prevents the development of typical Pythium leak necrosis on potato slice assay *in planta*. CRISPR–cas9 gene deletion studies of strain S1Bt23, S1Bt23Δ*phz*B and S1Bt23Δ*phz*F revealed the involvement of phenazines in antagonistic activity of strain S1Bt23 against *P. ultimum*.

## Results

### Taxonomic identification of strain S1Bt23

**Fig. 1:**
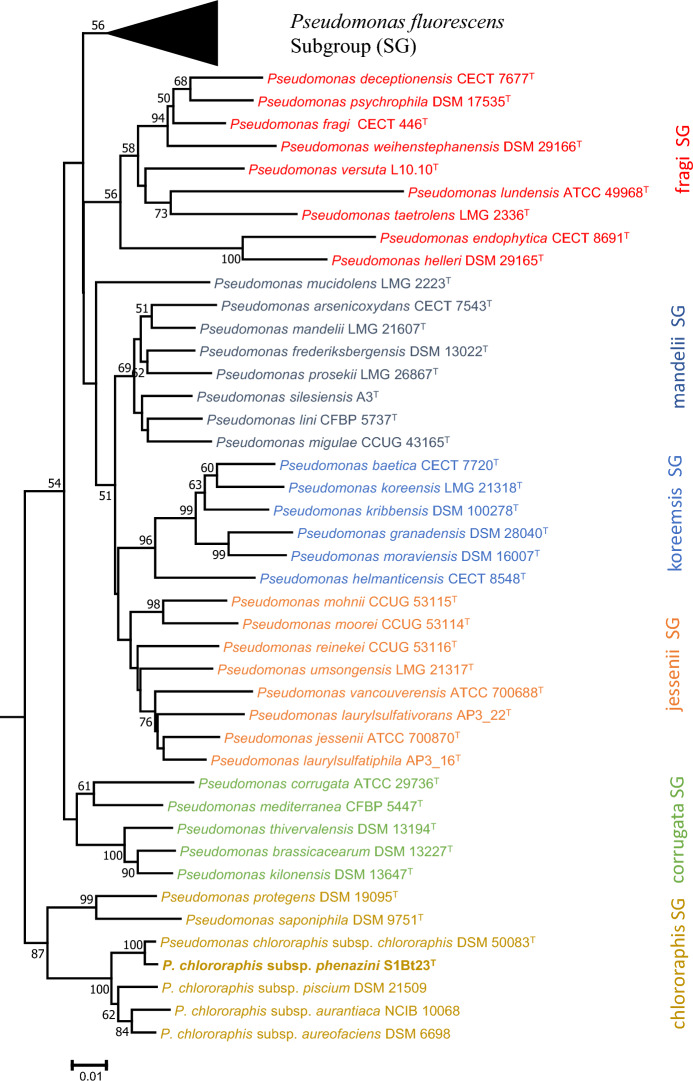
Neighbor-joining evolutionary tree of 16S rRNA-*gyr*B-*rpo*B-*rpo*D (2964 bp) concatenated nucleotide sequences showing strain S1Bt23 clustering distinctly within the *Pseudomonas chlororaphis* subgroup. The percentages of replicate trees in which the associated taxa clustered together in the bootstrap test (1000 replicates) are shown next to the branches. SG, subgroup.

BLAST and maximum likelihood-based (ML) phylogenetic analyses of the 16S rRNA gene sequence showed that strain S1Bt23 belonged to pseudomonads of the *P. fluorescens* group which consists of seven subgroups. The 16S rRNA sequence of S1Bt23, however, showed 98–99% similarity to 74 species that constitute the seven subgroups of the *P. fluorescens* group. This confirms a clear delineation at the genus-level but low resolution at the species level. Similarly, 16S rRNA ML-inferred evolutionary tree (Fig. S1) showed that strain S1Bt23 clustered with *P. chlororaphis* but the delineation of subgroups within the *P. fluorescens* group was fuzzy. For example, the *P. chlororaphis* subgroup (*P. chlororaphis*, *P. protegens* and *P. saponiphilia*) did not cluster tightly, confirming that 16S rRNA was a genus-level marker. Species-level identification was performed using the MLSA consisting of 16S rRNA (1328 bp), *gyr*B (397 bp), *rpo*B (666 bp) and *rpo*D (573 bp). A neighbor-joining tree (NJ) (Fig. 1) inferred using the concatenated 16S rRNA-*gyr*B-*rpo*B-*rpo*D (2964 bp) showed that strain S1Bt23 belonged to the *P. chlororaphis* subgroup and the expected six other subgroups of *P. fluorescens* group were accurately delineated. This analysis also showed that *P. chlororaphis* subsp. *chlororaphis* DSM 50083^T^ is the closest taxonomic relative of strain S1Bt23, suggesting that it belongs to the same species (Fig. 1). This affiliation was confirmed by phylogenomic analysis of 81 single-copy universal bacterial core genes (UBCG) of S1Bt23, representative members of the *P. chlororaphis* subgroup and that of each of the other six subgroups of *P. fluorescens* group (Fig. S2).

We used digital DNA–DNA hybridization (dDDH) and Average Nucleotide Identity (ANIb) to determine the genomic similarity between *Pseudomonas chlororaphis* S1Bt23, the 4 known *Pseudomonas chlororaphis* subspecies ( subsp. *chlororaphis*, subsp. *aureofaciens*, subsp. *aurantiaca* and subsp. *piscium*), the two other species of the *P. choloraphis* subgroup and representatives of each of the other 6 subgroups within the *P. fluorescens* group. The representative genome sequences of the other six subgroups exhibited dDDH (24.5–27.4%) and ANIb (79.2–82.1%) values that were below the respective species-level cut-offs of 70% and 95–96%, suggesting a distant relationship (Table 1). Also, *P. protegens* and *P. saponiphila* (members of the *P. chlororaphis* subgroup) had dDDH (29.1–29.2%) and ANIb (83.5 and 83.9%) values that were below the 70% and 95%, respectively (Table 1). The whole genome sequence of strain S1Bt23 and those of the reference subspecies of *P. c.* subsp. *aureofaciens* (60.6% and 94.3%), *P. c.* subsp. *aurantiaca* (59.6% and 94.5%) or *P. c.* subsp. *piscium* (58.0% and 94.1%) showed dDDH and ANIb values below the 70% and 95% thresholds (Table 1), suggesting that strain S1Bt23 cannot be taxonomically placed in the same species with these three subspecies. However, strain S1Bt23 exhibited dDDH and ANIb values of 74.9% (species cut-off = 70%) and 96.7% (cut-off 95–96%), respectively, with *P. chlororaphis* subsp. *chlororaphis* DSM50083^T^, an indication that these strains belong to the same species. While there is no subspeciation cut-off value for ANI values, the statistically established subspecies cut-off for dDDH is 79%^37,38^. Strain S1Bt23 and *P. chlororaphis* subsp. *chlororaphis* DSM 50083^T^ had a dDDH value of 74.9% (Table 1), which is below the cut-off value for subspecies delineation. We therefore propose that strain S1Bt23 represents an authentic novel subspecies of *Pseudomonas chlororaphis*.

**Table 1:** Whole-genome pairwise comparison of strain S1Bt23^T^ and type strains of the *Pseudomonas chlororaphis* subgroup and other *P. fluorenscens* subgroups based on in silico DNA–DNA hybridization (dDDH) and average nucleotide identity (ANI) values.

Bacterial species/strain (Genome accession #)	dDDH values (%)	ANIb value (%)	Genomic distance
*Pseudomonas chlororaphis* subgroup:			
* P. chlororaphis* subsp. *phenazini* subsp. nov. S1Bt23^T^	100	100	0
*P. chlororaphis* subsp. *chlororaphis* DSM 50083^T^ (CP027712)	**74.9 [71.9–77.7]**	**96.70**	0.0296
*P. chlororaphis* subsp. *aureofaciens* DSM 6698^T^ (CP027720)	60.6 [57.7–63.4]	94.32	0.0506
*P. chlororaphis* subsp. *aurantiaca* DSM 19603^T^ (CP027746)	59.6 [56.7–62.3]	94.52	0.0524
*P. chlororaphis* subsp. *piscium* DSM 21509^T^ (CP027707)	58.0 [55.2–60.7]	94.06	0.0552
*Pseudomonas protegens* CHA0^T^ (CP003190)	29.2 [26.9–31.7]	83.91	0.1464
*Pseudomonas saponiphil*a DSM 9751^T^ (FNTJ01)	29.1 [26.7–31.6]	83.57	0.1473
Other *P. fluorescens* subgroups:			
*Pseudomonas corrugata* LMG 2172^T^ (LT629798)	26.7 [24.3–29.2]	81.06	0.1622
*Pseudomonas jessenii* DSM 17150^T^ (NIWT01)	27.4 [25.0–29.9]	82.11	0.1576
*Pseudomonas koreensis* LMG 21318^T^ (LT629687)	27.0 [24.6–29.5]	81.75	0.1603
*Pseudomonas mandelii* LMG 21607^T^ (LT629796)	27.0 [24.6–29.5]	81.95	0.1602
*Pseudomonas fragi* NBRC 3458^T^ (BDAB01)	24.5 [22.2–26.9]	79.23	0.1782
*Pseudomonas gessardii* LMG 21604^T^ (FNKR01)	26.7 [24.4–29.2]	81.30	0.1619
*Pseudomonas fluorescens* NCTC10038^T^ (LS483372)	25.5 [23.2–28.0]	80.38	0.1704

Species-level cut-off value for dDDH and ANIb, respectively, were 70% and 96%.

Significant values are in bold.

**Fig. 2:**
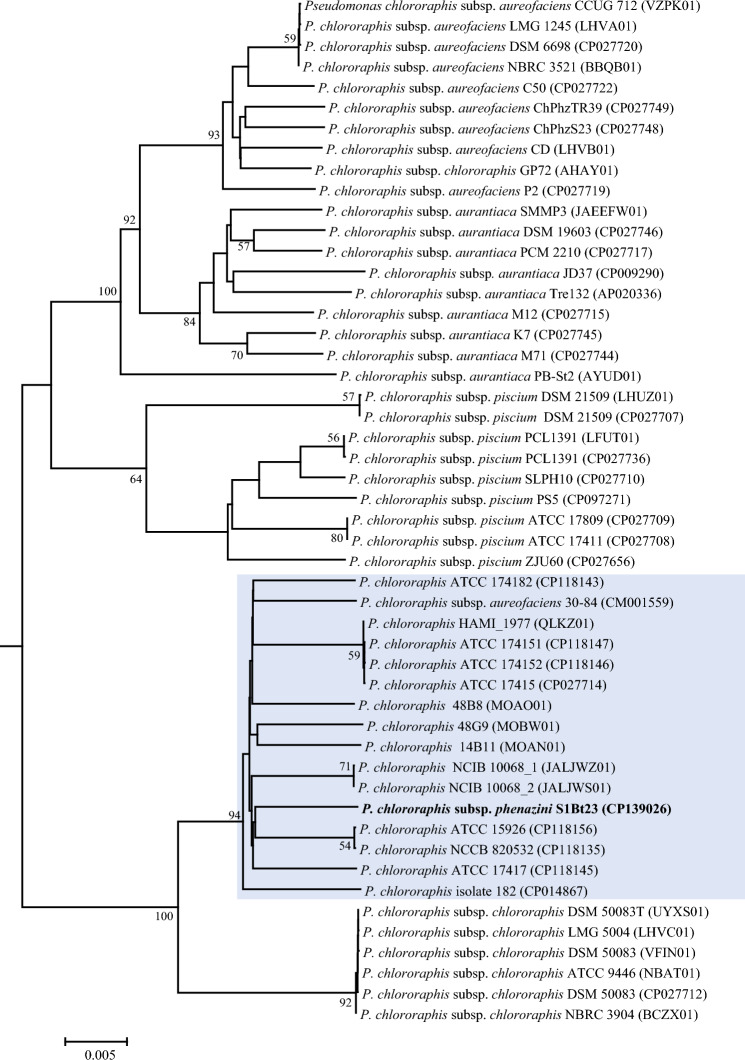
Genome-based TYGS-generated tree showing distinct evolutionary clustering of *Pseudomonas chlororaphis* subsp. *phenazini* strains (blue rectangle) relative to strains of the other *P. chlororaphis* subspecies. The TYGS algorithm classified S1Bt23 (in bold) and 15 NCBI strains as novel subspecies based on the cut-off for subspecies of 79.0%. Branch lengths are scaled according to the GBDP distance formula d5. Numbers above branches are GBDP pseudo-bootstrap support values > 60% from 100 replicates. The tree was rooted at the midpoint.

We computed dDDH values between strain S1Bt23 and all 135 draft and whole genome sequences of *P. chlororaphis* from NCBI to identify other genomes that might be genomically similar to the proposed novel subspecies. Fifteen NCBI genome sequences exhibited dDDH values of 83.6–86.0% (Table 2) with strain S1Bt23, suggesting that these strains belong to the new subspecies delineated in this study. These 15 whole genome sequences exhibited dDDH values, ranging from 57.9% (*P. c.* subsp. *piscium*) to 75.4% (*P. c.* subsp. *chlororaphis* ), below the subspecies cut-off of 79%, confirming their affiliation to the new proposed subspecies represented by strain S1Bt23. Fourteen of these whole genomes were classified in GenBank only as *P. chlororaphis* with no subspecies assignment. However, one genome (strain 30–84; GenBank accession # CM001559) currently classified as *P. chlororaphis* subsp. *aureofaciens* is an incorrect subspecies assignment based on our data. To confirm that these 15 genomes and S1Bt23 belong to a new subspecies, phylogenomic analysis was performed on the TyGS platform with a built-in algorithm for detection and identification of subspecies. The tree generated by TyGS shows a distinct evolutionary clustering of strain S1Bt23 and the 15 other genomes (Fig. 2; blue rectangle). In addition, the TyGS algorithm denoted that S1Bt23 and the other 15 NCBI genome sequences belong to the species *P. chlororaphis* but in a new subspecies.

**Table 2:** Digital DNA–DNA Hybridization (dDDH) values of strain S1Bt23 against 92 NCBI *Pseudomonas chlororaphis* genomes identified 15 strains as valid members of the new subspecies *phenazini* (subspeciation cut-off value = 79%).

Previous taxonomy/strain	Genome accession #	Digital DNA–DNA Hybridization (%)				
		S1Bt23	P.c.chlor	P.c.aureo	P.c.aurant	P.c.piscium
*Pseudomonas chlororaphis* ATCC 15926	CP118156	**86**.**0 [83**.**3–88**.**3%]**	75.0 [72.0–77.8%]	60.50 [57.7–63.3%]	59.9 [57.1–62.7%]	58.1 [55.3–60.9%]
*Pseudomonas chlororaphis* NCCB 820532	CP118135	**85**.**9 [83**.**2–88**.**2%]**	75.0 [71.9–77.7%]	60.50 [57.6–63.3%]	59.9 [57.0–62.6%]	58.1 [55.3–60.8%]
*Pseudomonas chlororaphis* ATCC 17417	CP118145	**85**.**5 [82**.**8–87**.**8%]**	75.3 [72.3–78.1%]	60.70 [57.8–63.5%]	59.8 [56.9–62.5%]	58.2 [55.4–61.0%]
*Pseudomonas chlororaphis* NCIB 10068_1^‡^	JALJWZ01	**85**.**5 [82**.**8–87**.**8%]**	75.4 [72.4–78.2%]	60.50 [57.7–63.3%]	59.8 [57.0–62.6%]	58.1 [55.3–60.9%]
*Pseudomonas chlororaphis* NCIB 10068_2^‡^	JALJWS01	**85**.**5 [82**.**8–87**.**8%]**	75.4 [72.4–78.2%]	60.50 [57.7–63.3%]	59.8 [57.0–62.6%]	58.1 [55.3–60.9%]
*Pseudomonas chlororaphis* ATCC 174182	CP118143	**84**.**6 [81**.**9–87**.**0%]**	75.1 [72.1–77.9%]	60.80 [58.0–63.6%]	59.8 [56.9–62.6%]	58.3 [55.5–61.1%]
*Pseudomonas chlororaphis* 48B8	MOAO01	**84**.**6 [81**.**8–87**.**0%]**	74.4 [71.4–77.2%]	60.10 [57.3–62.9%]	59.6 [56.8–62.4%]	58.1 [55.3–60.8%]
*Pseudomonas chlororaphis* 48G9	MOBW01	**84**.**4 [81**.**6–86**.**8%]**	74.9 [71.9–77.7%]	60.30 [57.5–63.1%]	59.9 [57.0–62.6%]	57.9 [55.1–60.6%]
*Pseudomonas chlororaphis* 14B11	MOAN01	**84**.**0 [81**.**2–86**.**4%]**	74.8 [71.8–77.6%]	60.20 [57.3–62.9%]	59.4 [56.6–62.2%]	58.1 [55.3–60.9%]
*Pseudomonas chlororaphis* ATCC 17415†	CP027714	**83**.**9 [81**.**1–86**.**3%]**	74.9 [71.9–77.7%]	60.10 [57.3–62.9%]	59.5 [56.6–62.2%]	58.0 [55.2–60.7%]
*Pseudomonas chlororaphis* ATCC 174152†	CP118146	**83**.**9 [81**.**1–86**.**3%]**	74.9 [71.9–77.7%]	60.10 [57.3–62.9%]	59.5 [56.7–62.3%]	58.0 [55.2–60.7%]
*Pseudomonas chlororaphis* ATCC 174151†	CP118147	**83**.**9 [81**.**1–86**.**3%]**	74.9 [71.9–77.7%]	60.10 [57.3–62.9%]	59.5 [56.7–62.3%]	58.0 [55.2–60.7%]
*Pseudomonas chlororaphis* HAMI_1977	QLKZ01	**83**.**8 [81**.**1–86**.**3%]**	74.9 [71.9–77.7%]	60.10 [57.3–62.9%]	59.4 [56.6–62.2%]	57.9 [55.1–60.7%]
*Pseudomonas chlororaphis* isolate 182	CP014867	**83**.**6 [80**.**8–86**.**0%]**	74.4 [71.3–77.2%]	60.30 [57.4–63.1%]	60.0 [57.1–62.7%]	58.2 [55.4–61.0%]
*P. chlororaphis subsp. aureofaciens* 30–84	CM001559	**84**.**5 [81**.**7–86**.**9%]**	74.8 [71.8–77.6%]	60.30 [57.5–63.1%]	59.5 [56.7–62.3%]	58.4 [55.5–61.1%]

Values in bold are > 79%, the subspeciation cut-off threshold. Numbers in square brackets are the model confidence interval. P.c.chlor, *P. chlororaphis* subsp. *chlororaphis* DSM 50083^T^ (CP027712); P.c.aureo, *P. c.* subsp. *aureofaciens* DSM 6698^T^ (CP027720); P.c.aurant, *P. c.* subsp. *aurantiaca* DSM 19603^T^ (CP027746); P.c.piscium, *P. c.* subsp. *piscium* DSM 21509^T^ (CP027707). ^‡^These two genome sequences might have been derived from the same strain NCIB 10068; and might have been derived from ATCC 17515.

### In silico detection of secondary metabolites and other virulence factors in strain S1Bt23

The PacBio de novo assembly of strain S1Bt23 produced high-quality complete whole-genome sequence of 6,755,322 nt, in length, in a single contig/scaffold. Annotation of the whole genome sequence of S1Bt23 using prokka revealed 6033 coding sequences, 16 rRNAs, 78 tRNAs, 1 tmRNA and 2 repeat_regions.

**Fig. 3:**
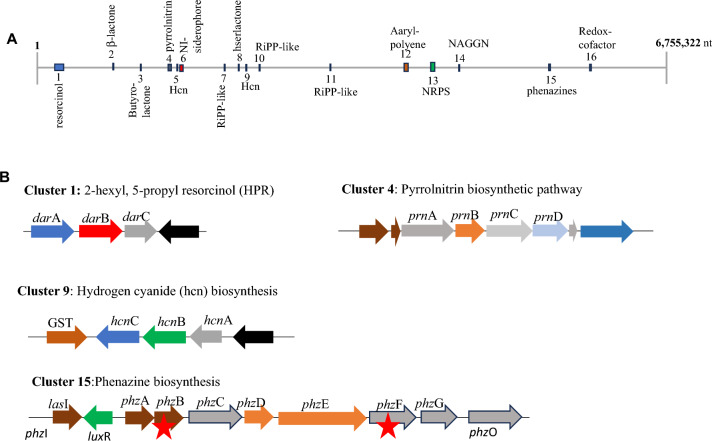
(**A**) Overview of the different genomic regions of potential secondary metabolites in *Pseudomonas chlororaphis* subsp. *phenazini* strain S1Bt23; and (**B**) Four complete biosynthetic clusters (cluster 1, 4, 9, and 15) previously reported to inhibit fungal/oomycete plant pathogens. NI-siderophore, NRPS-independent, IucA/IucC-like siderophores; NRPS, Non-ribosomal peptide synthetase; NAGGN, N-acetylglutaminylglutamine amide; RiPP-like, Other unspecified ribosomally synthesized and post-translationally modified peptide product (RiPP) and Hcn, hydrogen cyanide; GTS, glutathione S-transferase. The exact coordinates of the regions can be found in Figure S5. Red stars indicate the genes deleted (*phz*B and *phz*F) in our study.

The whole-genome sequence of S1Bt23 was scanned for potential secondary metabolite biosynthesis gene clusters using antiSMASH algorithm. Figure 3A summarizes the detected clusters including betalactone, butyrolactone, pyrrolnitrin, NI-siderophore, hserlactone, arylpolyene, NRPS, hydrogen cyanide, NAGGN, phenazine and redox cofactor. Comparison of these clusters with the MIBiG repository of clusters of known function identified several antimicrobial molecules or genes. Figure 3B shows the presence of four complete biosynthetic clusters previously implicated in biological control of fungal plant pathogen. These include clusters required for biosynthesis of resorcinol (*dar*ABC), pyrrolnitrin (*prn*ABCD), hydrogen cyanide (*hcn*ABC) and a putative Hcn ABC transporter ATP-binding protein; and phenazine (*phz*ABCDEFG). MIBiG also identified phenazines to the closest in the database which was pyocyanine from *Pseudomonas aeruginosa* PAO1. Region 4 showed a cluster with 100% similarity to pyrrolnitrin while region 6 was identified to be 100% hydrogen cyanide. The complete phenazine cluster detected (Fig. 3B) had a *phz*O which might be required to convert phenazine-1-carboxylic acid (PCA) to 2-hydroxphenazine-1-carboxylic acid (2-OH-PCA) and into 2-hydroxphenazine (2-OH-PHZ). Prism4 software predicted the structure of a product of the phenazine cluster to be PCA. This suggests that the core genome of strain S1Bt23 is rich in potentially bioactive secondary metabolites.

Genome mining for other virulence factors using the VFDB tool^39^ revealed a repertoire that can be divided into 20 classes including adherence, antimicrobial activity, antiphagocytosis, enzyme, iron uptake, protease, quorum sensing, regulation, secretion system, toxin, biofilm formation, cell surface components, efflux pump, glycosylation system, immune evasion, invasion, lipid and fatty acid metabolism, magnesium uptake, serum resistance and stress adaptation. Adherence-related genes consisted of flagella, type IV pili biosynthesis, and twitching motility related proteins were similar to those of *P. c. subsp. chlororaphis* DSM 50083^T^. No gene involved in biosurfactant, e.g. rhamnolipid biosynthesis was evident. Subtle differences in iron uptake were observed between S1Bt23 and *P. c. chlororaphis* DSM 50083^T^ with the presence of *pvd*D in the former but not in the latter. In contrast, a pyochelin-related gene *pch*F was absent in S1Bt23 but present in strain DSM50083. With respect to immune evasion, four copies of the *pse*B-like gene which is involved in capsule formation in *Acinetobacter* were found in the genome of S1Bt23. In stress adaptation, S1Bt23 had a unique manganese transport system similar to that of Neisseria but absent in Pcc DSM 50083. The virulence factors detected indicates potential nonpathogenicity of S1Bt23 when compared to *Pseudomonas aeruginosa* and *Pseudomonas syringae*. The VFDB algorithm^39^ confirmed the presence of the complete phenazine biosynthetic cluster, *phz*ABCDEFG, as well as all the three genes (*hcn*ABC) required for the production of hydrogen cyanide, a potent antifungal molecule.

### CRISPR/Cas9 *phz* gene knockouts; and TLC and HPLC analyses

**Fig. 4:**
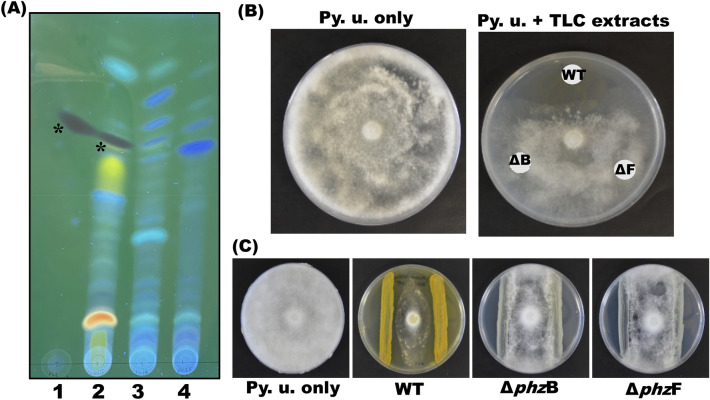
(**A**) Thin layer chromatography silica60 plate, under ultra-violet light, showing the loss of phenazine production (*dark gray band) in the pAKanCRISPR–cas9 mutants of S1Bt23: 1, positive control (synthetic phenazine; 20 µl of 1 mg/ml stock); 2, S1Bt23 wild type; 3, S1Bt23Δ*phz*B; and 4, S1Bt23Δ*phz*F mutants (right); (**B**) TLC extracts from WT inhibited growth of *Pythium ultimum* (Py. u.) but not those from mutants (ΔB or ΔF); and (**C**) WT inhibited Py. u. growth in dual culture assay but not Δ*phz*B or Δ*phz*F mutants.

S1Bt23 inhibited radial mycelial growth of *Pythium ultimum* LevI 805 in in vitro dual cultures on GCY medium with rates ranging from 60.2.0 to 66.7.0%. To test the direct involvement of phenazines in the antifungal activity of S1Bt23, the *phz*B and *phz*F genes were deleted using pACRISPR and pCasPA system^40^ that is based on resistance to ampicillin as the selective marker. However, initial antibiotic sensitivity tests indicated that S1Bt23 (wild type) is resistant to ampicillin, but susceptible to kanamycin (Fig. S3). We, therefore, modified the backbone of the plasmid by replacing ampicillin resistance gene sequence with that of kanamycin resistance from a donor plasmid, pGWN2, resulting in pAKanCRISPR and transformed it into *E. coli* DH5alpha. The new plasmid and pCasPA were used to create mutants of S1Bt23 by deleting the *phz*B (S1Bt23Δ*phz*B) or *phz*F (S1Bt23Δ*phz*F) genes. The deletions of the *phz*B and *phz*F genes were confirmed by PCR (Fig. S4). On LB medium, the mutants appear to have a low orange pigmentation, a signature characteristic of the wild type, S1Bt23 (Fig. S5). TLC and HPLC analyses were performed to investigate the effects of *phz*B or *phz*F gene deletions on phenazine biosynthesis in S1Bt23 and its mutants. UV light visualization of the developed TLC plates revealed the loss of the dark gray band corresponding to phenazine in both mutants at relatively the same retention factor as the pure synthetic phenazine (Fig. 4A). The total metabolite extracts or TLC plate-extracted dark gray band alone inhibited the mycelia growth of *P. ultimum* (Fig. 4B). Furthermore, HPLC analysis of the extracts of the two mutants confirmed complete loss of phenazine production following *phz*B or *phz*F deletion as the characteristic peak at a retention time of 10.78 min observed for S1Bt23 (WT) or synthetic PCA was absent (Fig. S6).

### Antagonistic activity of S1Bt23 and its mutants against P. ultimum

To test whether phenazine production is involved in the antagonism of *P. ultimum* by strain S1Bt23, dual culture assays were performed with S1Bt23 (WT), S1Bt23Δ*phz*B (Δ*phz*B) and S1Bt23Δ*phz*F (ΔphzF) mutants. As expected, wildtype S1Bt23 showed potent antagonistic activity against *P. ultimum* (Fig. 4C). The deletion of *phz*B or *phz*F in S1Bt23 abrogated this antagonistic activity (Fig. 4C), indicating the involvement of phenazines. The streaked *phz*B and *phz*F mutants could not inhibit *P. ultimum* but seemed to act as physical barrier for the growth progression of the oomycete (Fig. 4C).

**Fig. 5:**
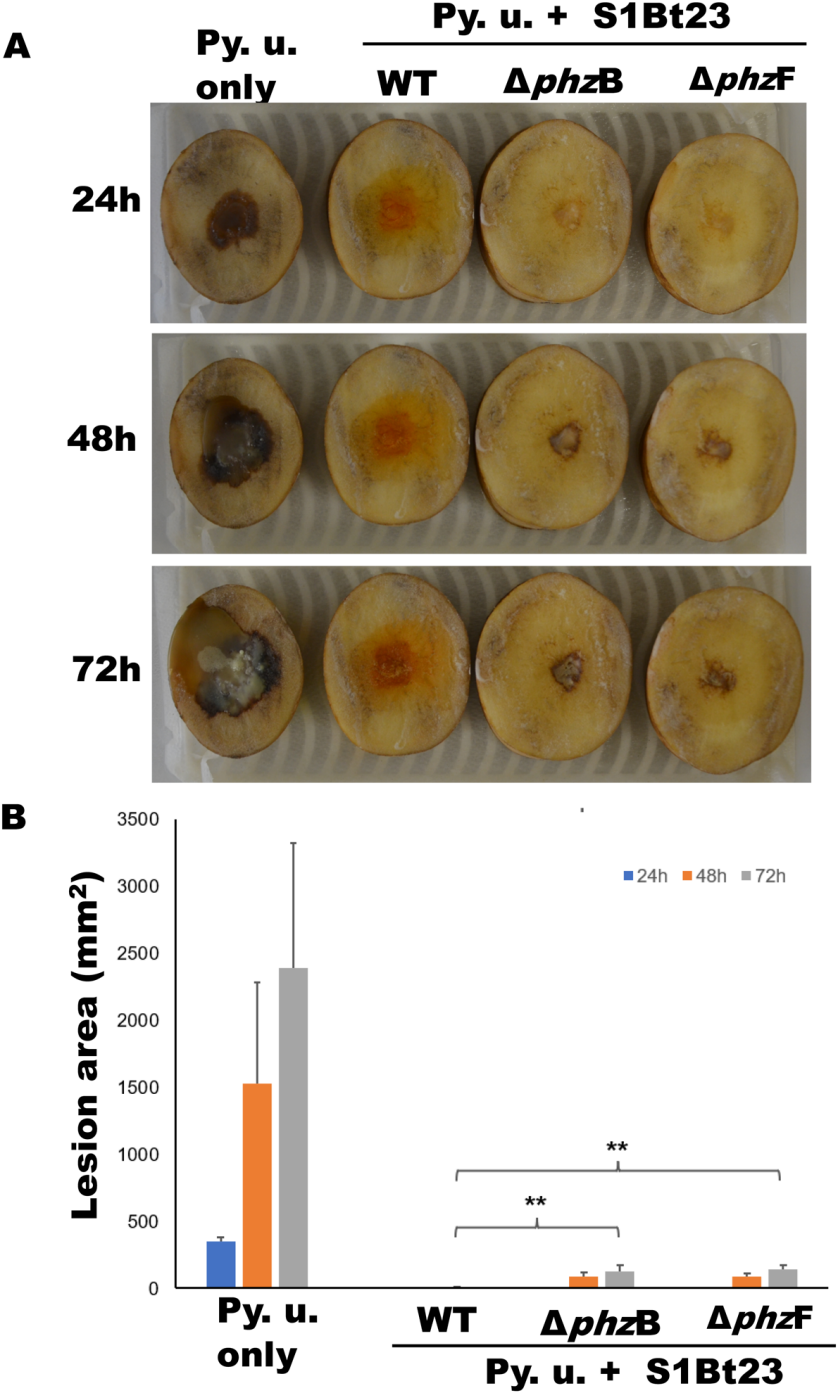
(**A**) *Pseudomonas chlororaphis* subsp. *phenazini* strain S1Bt23 (WT) shows significant protection of potato tuber slices against *Pythium ultimum* (Py. u.) compared with Δ*phz*B or Δ*ph*zF mutants. No necrosis was observed on potato tuber slices treated with bacteria alone. (**B**) Statistical analysis of area of lesions calculated using mean diameter of the lesion [area = π(*d*/2)^2^. Statistical analysis using one-way ANOVA. ** indicates *p* value < 0.01.

The in vivo tuber slice inoculation bioassay was also used to investigate the potential bioprotection of potato by S1Bt23 against *P. ultimum*, the Pythium leak disease pathogen. Figure 5A shows the progression of Pythium leak disease symptoms over a 72-h period. While treatments with *P. ultimum* alone showed highly significant (*p* < 0.001) necrotic areas of tuber slices, no or very little necrotic areas were recorded when slices were treated with S1Bt23 (Fig. 5B). Also, the necrotic surface area of S1Bt23 (WT)-treated tuber slices was statistically lower than those of the mutants (Fig. 5B). Although the necrotic areas of tuber slices treated in *Pythium* + mutants were significantly lower than those treated with *Pythium* alone, the characteristic watery black soft symptoms were similar, and were not observed in wild type with the treatments. The low but observable necrotic lesions on potato tuber slices inoculated with *Pythium* + mutants could be attributed to other active compounds, e.g. pyrrolnitrin, hydrogen cyanide, which might have been facilitated by direct contact with the applied bacterial biofilm.

### Morphology, phenotypic and biochemical traits

Cells of S1Bt23 were rod-shaped, not helical, 0.6–0.8 × 1.8–2.3 µm (Fig. S7). Table 3 summarizes the phenotypic characteristics that distinguish S1Bt23, representative of the new subspecies, from the other four known subspecies. S1Bt23 can be differentiated from subsp. *chlororaphis* LMG 50083^T^, subsp. *aureofaciens* LMG 1245 and subsp. *piscium* DSM 21509 by the utilization of D-galactose, and from subsp. a*urantiaca* LMG 21630 by the assimilation of uridine. The optimum growth temperature range of S1Bt23 was 28–37 °C. Strain S1Bt23 grew in 2% and 4% salt after 24 h and 48 h but in 8% only after 48 h. It grew at pH 5–6. The major cellular fatty acid peaks (Table S2) were C_16 : 1_ ω6c/C_16 : 1_ ω7c (summed feature 3), C_16 : 0_, C_18 : 1_ ω7c/C_18 : 1_ ω6c (summed feature 8), C_12 : 0_ 3-OH, C_10 : 0_ 3-OH, C_12 : 0_ 2-OH, and C17 : 0 cyclo. The major polar lipids of S1Bt23 were phosphatidylethanolamine (PE), diphosphatidylglycerol (DPG) and phosphatidylglycerol (PG) (Fig. S8). Minor amounts of lipid and phospholipids were also detected (Fig. S8). The fatty acid and polar lipid profiles are consistent with those of members of the genus *Pseudomonas*.

**Table 3:** Phenotypic characteristics differentiating the new subspecies from the other four *Pseudomonas chlororaphis* subspecies: (1) subsp. *phenazini* S1Bt23^T^; (2) subsp. *chlororaphis* LMG 5004^T^; (3) subsp. *aureofaciens* LMG 1245^T^; (4) subsp. *aurantiaca* LMG 21630^T^; and (5) subsp. *piscium* DSM 21506^T^. S1Bt23, can be differentiated from LMG 5004^T^, LMG 1245^T^ and DSM 21509^T^ by utilizing D-Galactose; and from LMG 21630 by assimiliating uridine.

Reaction or substrate	1	2	3	4	5
API 20NE:					
Reduction of nitrate to nitrites	**−**	**+**	**−**	**−**	**+**
Phenylacetic acid assimilation	+	−	+	+	+
Indole production (L-Tryptophan)	w	w	w	−	w
Biolog PM1 & PM2A:					
D-Galactose	+	−	−	+	−
D-Trehalose	+	−	+	+	+
D-Mannose	+	−	+	+	+
L-Lactic Acid	−	+	+	+	+
Formic Acid	−	−	+	−	+
a-D-Glucose	+	−	+	+	+
Sucrose	+	−	+	+	+
Uridine	+	−	+	−	−
a-Hydroxy Glutaric Acid-y-Lactone	−	+	+	−	+
Adenosine	+	−	+	+	+
Methyl Pyruvate	+	−	+	+	+
Phenylethylamine	+	−	+	+	+
2-Aminoetha	+	_−_	+	+	+

+, positive reaction; −, negative reaction; and w, weak reaction.

## Discussion

This study reports the taxonomic characterization of a novel strain (S1Bt23) of *P. chlororaphis* isolated from Canadian woodland soil using a suite of whole genome-based parameters coupled with MLSA and phenotypic data to conclude that it belongs to an undescribed subspecies. Also, we demonstrated its potent antagonistic activity against *P. ultimum* and used the CRISPR–cas9 knockout technology to show the involvement of phenazines.

Accurate identification of bacterial strains at the species and subspecies levels is key to better understanding their diversity and evolution^41–43^ and to predicting potential applications based on known relatives. In the last decade, this has been facilitated by advances in high-throughput sequencing and bioinformatics tools that allow the use of whole genome sequences for identification of bacterial strains^41,43^. In this study, we exploited the high resolution power of genomic data (dDDH, ANIb and phylogenomics) to identify strain S1Bt23 at the species and subspecies levels. With species cut-offs of 70% and 96% for dDDH (74.9%) and ANIb (96.7%) values, respectively, strain S1Bt23 was accurately assigned to *P. chlororaphis*, with the type strain, *P. c.* subsp. *chlororaphis* DSM 50083^T^ showing the highest taxonomic relationship. It is worth noting that the representatives of the other subspecies of *P. chlororaphis* showed dDDH (58.0–60.9%) and ANIb (94.1–94.5%) values below the cut-off threshold with strain S1Bt23 or *P. c.* subsp. *chlororaphis* DSM 50083^T^, suggesting that these strains do not belong to the same species. *P. c.* subsp. *aureofaciens* and *P. c.* subsp*. aurantiaca* were initially classified as *Pseudomonas aureofaciens*^44^ and *Pseudomonas aurantiaca*^45^ and confirmed by whole genome sequence data in our study. Peix et al.^34^ reclassified these two species as *P. chlororaphis* using wet-lab DNA–DNA hybridization (wDDH) values. Although the wDDH technique has long been the gold standard for classification purposes^46^, it is now known to show inherent drawbacks, such as irreproducibility, high error and failure to produce accumulative databases^43,47,48^. Taxonomic classification of bacteria based on whole genome sequence is now the new gold standard that minimizes most of the pitfalls of wDDH^41,43,49,50^. Also, a dDDH value of 74.30% (71.3–77.1%) computed between *P. c.* subsp. *aureofaciens* DSM 6698^T^ (CP027720) and *P. c.* subsp. *aurantiaca* DSM 19603^T^ (CP027746) suggests that these strains belong to a new species but *P. chlororaphis* and each represents a distinct subspecies. Also, *P. c. subsp. piscium* had dDDH values (cut-off = 70%) of 63.20% [60.3–66%] and 62.70% [59.8–65.5%] with the respective whole genome sequences of *P. c.* subsp. *aureofaciens* and *P. c.* subsp*. aurantiaca,* suggesting their affiliations to different species. A genome-based taxonomic review of these subspecies is warranted.

Our dDDH data (74.9%) indicated that strain S1Bt23 is a true member of *Pseudomonas chlororaphis* but a new subspecies based on the subspecies cut-off value of 79.0%^37,38^. This subspecies threshold based on dDDH is routinely used to delineate bacterial subspecies due to its consistency and reproducibility across laboratories^51–53^. We also analyzed all the 135 NCBI whole genome entries and identified 15 strains that had dDDH values of 83.9–86.0% with strain S1Bt23 but exhibited only 74.4–75.0% homology with *P. c*. subsp. *chlororaphis* DSM 50083^T^ or 58.0–60.9% with the other subspecies. These data suggest that these 15 strains belong to the newly proposed subsp. *phenazini*. The 15 strains and S1Bt23 (new subspecies) were isolated only in Canada and USA suggesting North American ecosystem-specific microbes or could be attributed to intense research on this species in this region. In addition, all but one of these strains were identified in GenBank as *P. chlororaphis* without subspecies assignment. Strain 30–84 (CM001559), a physiologically and genetically well studied biological strain^54–56^ classified as *P. c. subsp. aureofaciens* should be transferred to the subspecies *phenazini* based on the data presented in our study. The uniqueness of members of this new subspecies, as represented by S1Bt23, was also evident in the phenotypic data by utilizing D-galactose, D-trehalose, and D-mannose as sole carbon sources but not its closest taxonomic relative, *P. c.* subsp. *chlororaphis*. These two subspecies could also be differentiated by pigment production. Strain S1Bt23 secretes an orange pigment in contrast to the green coloration of *P. c.* subsp*. chlororaphis* DSM 50083^T^ on the same medium. Based on all these data, strain S1Bt23 represents a new subspecies named *phenazini* (referring to the ability to produce phenazine).

Mining the whole genome sequence of strain S1Bt23 for antifungal secondary metabolite biosynthetic clusters and other virulence factors revealed an arsenal that might be deployed to enhance its fitness in competitive ecosystems. MIBiG identification of 2-hexyl, 5-propyl resorcinol (region 1), β-lactone (region 2), pyrrolnitrin (region 4), hydrogen cyanide (region 6), and phenazines (region 15) (Fig. 3) suggests that S1Bt23 may have a robust ecological fitness. The phenazine cluster was found to be complete consisting of *phz*ABCDEFG including phzI gene which is required for the synthesis of N-aceyl-homoserine lactone (N-aceyl-HSL) and a phzO gene involved in the conversion of PCA to 2-hydroxphenazine-1-carboxylic acid (2-OH-PCA) which can be spontaneously converted consequently to 2-hydroxphenazine (2-OH-PHZ). It is therefore highly probable that strain S1Bt23 also produces 2-OH-PCA and/or 2-OH-PHZ, one of which could be represented by a second peak lagging that of PCA (depicted with an asterisk *; Fig. S6) detected by HPLC analysis. Additional work will be done to confirm that 2-OH-PCA and/or 2-OH-PHZ are produced by strain S1Bt23. Most of the secondary metabolites identified in this study are reported to have antimicrobial properties.

Given this comprehensive arsenal, we hypothesized that strain S1Bt23 could be a potent biological control agent against plant pathogens and investigated its effect against *P. ultimum*, a pathogen of several important agricultural crops such as tomato, potato, soybean, wheat and corn^57–61^. Our data, in vitro and on potato slices, determined that strain S1Bt23 is a potent biological agent against *P. ultimum* and the mechanism(s) involved were unknown.

Phenazines have been implicated in the biological control of several important fungal and oomycetous plant pathogens^25,62,63^. Phenazines showed significant antifungal activities against *P. ultimum*^64^*.* Significant reductions in damping-off disease symptoms caused by *P. ultimum* were achieved by the application of phenazine-producing variants of wild-type *Pseudomonas fluorescens* in heavily infested soil conditions^65^. The redox-active nature of phenazines by reducing molecular oxygen to create toxic reactive oxygen species explains their broad-spectrum capability^66^. Phenazine compounds have been reported to play a role in iron acquisition by reducing soil Fe^3+^ to Fe^2+^ (more soluble) as well as facilitating NADH reoxidation and pyruvate utilization in oxygen-limiting environments^67,68^. Here, we developed and used a modified pACRISPR–cas9 system, denoted as pAKanCRISPRK-cas9, by incorporating the kanamycin resistance gene as a selective marker instead of ampicillin (initial marker) which S1Bt23 is resistant to. Without this approach, it would not have been possible to deploy the pACRISPR–cas9 system to elucidate whether phenazines are involved in the inhibitory effect of S1Bt23 against *P. ultimum*. We investigated the potential involvement of phenazines in the inhibitory effects of strain S1Bt23 on *P. ultimum* using the pAKanCRISPR–Cas9 gene deletion system. The inhibitory effects against *P. ultimum* in vitro and bioprotection of potato slices were lost after deletion of either *phz*B (mutant S1Bt23Δ*phz*B) or *phz*F (mutant S1Bt23Δ*phz*F) compared to the wild type. These CRISPR–Cas9 gene deletion studies strongly suggest the involvement of phenazines in the antagonistic activity of strain S1Bt23 against *P. ultimum*. *phz*A, *phz*B, and *phz*G are reported to be required for the conversion of trans-2,3-dihydro-3-hydroxyanthranilic acid (DHHA) to PCA^69^. In addition, PhzF is an essential isomerase required for the condensation of two trans-2,3-dihydro-3-hydroxyanthranilate to phenazine ring^69^. As such, disruption of any of these genes, *phz*B in this study, will negatively impact the antifungal/anti-oomycete ability of the strain. McDonald et al.^70^ reported a fourfold decrease in PCA production when extract containing PhzC-G was supplemented with only PhzB and eightfold if both PhzA and -B were omitted. Incubation of extracts containing PhzB or PhzAB with ADIC did not lead to the production of PCA or other new products and concluded that these proteins have an important but nonessential role in phenazine biosynthesis, potentially in the stabilization of a hypothetical PCA-synthesizing multienzyme complex^71^. Due to the high homology (80% amino acid identity)^71^ of the PhzA and PhzB proteins, they are often referred as PhzA/B and are reported to catalyze the formation of tricycle of the phenazine molecule^66^. The results of our study suggest that although PhzA and PhzB are compositionally similar both or, at least, *phz*B are required for phenazine biosynthesis as evidenced by the deletion of *phz*B alone in S1Bt23.

Based on the TLC and HPLC data, the orange-colored phenazine (PCA) secreted by strain S1Bt23 is structurally and functionally different from pyocyanin, a blue-colored phenazine produced by the opportunistic human pathogen *Pseudomonas aeruginosa*. Pyocyanin is one of the virulence factors of *P. aeruginosa* in human pathogenesis^72,73^. Its zwitterion nature facilitates biological cell membrane permeability^74^ leading to disruption of cultured human epithelial cells^75^ resulting in premature senescence of fibroblasts and neutrophil apoptosis^76–78^. No similar negative reports of the effects of PCA on human cells are publicly available to the best of our knowledge but potential risks cannot be ruled out.

A bacterial strain S1Bt23 isolated from an undisturbed woodland soil in Aylmer, Québec, Canada, was found to inhibit the growth of *P. ultimum*, in vitro, and to protect potato tuber slices from the development of typical Pythium leak necrotic symptoms. Using pAKanCRISPR–Cas9 gene deletion system, we demonstrated that phenazines are involved in the antagonistic activity of strain S1Bt23 against *P. ultimum*. Based on polyphasic analysis of 16S rRNA, multilocus sequences, genome-based DNA–DNA hybridization and BLAST-based average nucleotide identity values as well as biochemical/physiological characteristics, we taxonomically conclude that this strain represents a novel subspecies within *P. chlororaphis* and propose the name *Pseudomonas chlororaphis* subsp. *phenazini* subsp. nov. with S1Bt23^T^ , the type strain. Further work will determine whether the antagonistic activity of strain S1Bt23 is broad-spectrum by testing other oomycetes and fungal plant pathogens.

### Description of *Pseudomonas chlororaphis* subsp. *phenazini* subsp. nov.

*Pseudomonas chlororaphis* subsp. *phenazini* (phe’na’zini. N.L. gen. n. *phenazini*, of phenazine, referring to the ability to produce phenazine).

The characteristics of this new subspecies are as described for species *P. chlororaphis* (Guignard & Sauvageau, 1894) Bergey & al. 1930 extended with data generated from our study. Strain S1Bt23 grew at 25–37 °C (optimum, 28–37 °C) but not at 5 °C, 15 °C or 40 °C. The strain grew at pH 5–6. Strain S1Bt23 grew in 2% and 4% salt after 24 h and 48 h but in 8% only after 48 h. Cells are Gram-reaction-negative, aerobic and rod-shaped with mean size of 0.60–0.80 × 1.8–2.3 µm. Colonies are circular (mean diameter of 4 mm), convex with regular margins on KB medium after 48 h. Strain S1Bt23 grows well on KB, LB, nutrient agar and tryptic soya agar. Orange pigment is produced. Nitrate reduction is negative. Based on Biolog (PM1 and PM2A) assays, S1Bt23 utilizes l-arabinose, N-acetyl-d-glucosamine, d-saccharic acid, succinic acid, d-galactose, l-aspartic acid, l-proline, d-alanine, d-trehalose, d-mannose, d-serine, d-sorbitol, glycerol, l-fucose, d-gluconic acid, d-mannitol, l-glutamic acid, d-glucose-6-phosphate, d-galactonic acid-γ-lactone, D,l-malic acid, d-ribose, tween 20, d-fructose, acetic acid, α- d-glucose, l-asparagine, d-aspartic acid, d-glucosaminic acid, 1,2-propanediol, tween 40, α-keto-glutaric acid, sucrose, uridine, l-glutamine, m-tartaric acid, d-glucose-1-phosphate, d-fructose-6-phosphate, tween 80, adenosine, glycyl-l-aspartic acid, citric acid, m-inositol, d-threonine, fumaric acid, propionic acid, mucic acid, inosine, glycyl-l-glutamic acid, tricarballylic acid, l-serine, l-threonine, l-alanine, l-Alanyl-glycine, methylpyruvate, l-malic acid, glycyl-l-proline, p-hydroxyphenyl acetic acid, m-hydroxy phenyl acetic acid, tyramine, pyruvic acid, phenylethylamine, 2-aminoetha, gelatin, laminarin, pectin, d-arabitol, γ-aminobutyric acid, butyric acid, capric acid, caproic acid, citramalic acid, d-glucosamine, 4-hydroxybenzoic acid, β-hydroxybutyric acid, itaconic acid, 5-keto-d-gluconic acid, d-lactic acid, methyl ester, malonic acid, oxalomalic acid, quinic acid, sorbic acid, l-alaninamide, N-acetyl-l-glutamic acid, l-arginine, glycine, l-histidine, hydroxy-l-proline, l-isoleucine, l-leucine, l-ornithine, l-phenylalanine, l-pyroglutamic acid, l-valine, D,l-carnitine, D,l-octopamine, putrescine and dihydroxy acetone. Strain S1Bt23 does not utilize dulcitol, d-glucuronic acid, D,l-α-glycerol-phosphate, d-xylose, l-lactic acid, formic acid, l-rhamnose, maltose, d-melibiose, thymidine, α-Keto-butyric acid, α-methyl-d-galactoside, α- d-lactose, lactulose, α-hydroxy glutaric acid-γ-Lactone, α-hydroxy butyric acid, β-Methyl-d-glucoside, adonitol, maltotriose, 2-Deoxy adenosine, bromo succinic acid, glycolic acid, glyoxylic acid, d-cellobiose, acetoacetic acid, N-Acetyl-β d-mannosamine, mono methylsuccinate, d-malic acid, d-psicose, l-lyxose, glucuronamide, l-galactonic acid -γ -lactone, d-galacturonic acid, chondroitin sulfate C, α-cycloextrin, β-cyclodextrin, γ-cyclodextrin, dextrin, glycogen, inulin, mannan, N-acetyl-d-galactosamine, N-acetyl-neuraminic acid, β-d-allose, amygdalin, d-arabinose, l-arabitol, arbutin, 2-deoxy-d-ribose, I-erythritol, d-fucose, 3–0-β-d-galacto-pyranosyl-d-arabinose, gentiobiose, l-glucose, lactitol, d-melezitose, maltitol, α-methyl-d-glucoside, β-methyl-d-galactoside, 3-methyl glucose, β-methyl-d-glucuronic acid, α-methyl-d-mannoside, β-methyl-d-xyloside, palatinose, d-raffinose, salicin, sedoheptulosan, l-sorbose, stachyose, d-tagatose, turanose, xylitol, N-acetyl-d-glucosaminitol, δ-amino valeric acid, citraconic acid, 2-hydroxbenzoic acid, γ-hydroxy butyric Acid, α-keto valeric acid, melibionic acid, oxalic acid, d-ribono-1,4-lactone, sebacic acid, succinamic acid, d-tartaric acid, l-tartaric acid, acetamide, l-homoserine, l-lysine, l-methionine, sec-butylamine, 2,3-butanediol, 2,3-butanone, 3-hydroxy 2 -butanone.

The type strain is S1Bt23^T^ (= CFBP 9180 = LMG 33497), isolated from soil collected in Aylmer, Quebec, Canada (45° 22′ 48.21″ N, 75° 48′ 25.52″ W). The DNA G + C content of S1Bt23^T^ is 62.90 mol%.

## Materials and methods

### Bacterial strains, plasmids, *Pythium* isolate, and DNA extraction

The isolation of strain S1Bt23 is described in Tchagang et al.^28^. The bacterial strains and plasmids used or mutants generated in this study are listed in Table S3. All strains were grown in Luria–Bertani (LB) Broth (Sigma, Canada), unless otherwise indicated, at 28–30 °C for strain S1Bt23 and its mutants, while *Escherichia coli* was cultured at 37 °C. Table S3 also has some phenotypic characteristics of the strains. Antibiotic concentrations used were 150 µg/mL carbenicillin (S1Bt23 and *E. coli*), 50 µg/mL kanamycin (S1Bt23 and *E. coli*), and tetracycline at 100 µg/mL or 20 µg/mL for S1Bt23 or *E. coli*, respectively.

Overnight grown cultures were used for downstream processing such as DNA extraction. DNA extraction and quantification for PCR amplification and multigene or genome sequencing were performed as previously reported^21^.

*Pythium ultimum* isolate LevI 805 was kindly provided by the laboratory of Dr. André Levesque (retired), at the Ottawa Research and Development Center, Ottawa, Ontario, Canada. *Pythium ultimum* isolate was cultivated on potato dextrose agar (Difco Becton Dickinson, Canada).

### Genome sequencing and GenBank downloads

Whole genome sequencing of strain S1Bt23 was performed using a single Molecule, Real-Time (SMRT) cell on a PacBio RSII sequencer (Pacific Biosciences, Canada) at the Innovation Centre (Génome-Québec, Montréal, Canada). This generated a total of 47,700 raw subreads of average length of 8209 bp, and de novo assembly performed using PacBio’s SMRT Link software (HGAP4 with a quality cutoff of 30). Alignments were then corrected prior to the implementation of Falcon tool to generate contigs. Circlator tool^79^ was used for circularization. Unless otherwise noted, default parameters were used for all software. The genome sequence was annotated with prokka 1.14.5^80^.

One hundred and thirty-five draft and whole-genome sequences identified as *P. chlororaphis* (Table S1) were downloaded from the GenBank database at the National Center for Biotechnology Information (NCBI, http://www.ncbi.nlm.nih.gov/genome/).

### Phylogenetic analysis, genome comparison, and phylogenomics

16S rRNA and MLSA (concatenated 16S rRNA-*gyr*B-*rpo*B-*rpo*D, 2964 bp long) sequencing, BLAST analysis and phylogenies of S1Bt23 and all the 76 species and subspecies of the *P. fluorescens* group were performed as previously reported^21,28^. Bootstrapping with 1000 replicates was used to validate the robustness of the branches.

Genome-to-genome distance calculator ver 3.1 (dDDH; species cut-off 70%; Meier-Kolthoff et al.^81^) and BLAST-based ANI (ANIb; cutoff 95–96%; Richter et al.^82^) were used to genomically compare S1Bt23 with all the species of the *P. chlororaphis* subgroup and representatives of the 7 other subgroups of the *P. fluorescens* group^42^. Since the dDDH analysis can reliably assign strains to the subspecies level (cut-off of 79%), it was used to compare strain S1Bt23 with all the 135 NCBI *P. chlororaphis* genomes. The dDDH tool is based on the principle of Genome BLAST distance phylogeny (GBDP).

Phylogenomic analysis was performed between S1Bt23, the four subspecies of the *P. chlororaphis* subgroup and representatives of the 7 other subgroups of the *P. fluorescens* group using the Type (Strain) Genome Server (TyGS)^37,38^ by implementing the MASH algorithm^83^. The resulting intergenomic distances were piped into FASTME 2.1.4^84^ to infer evolutionary trees with subtree pruning and regrafting (SPR) using the 100 pseudo-bootstrap postprocessing option. Trees were rooted at the midpoint^85^. The built-in parameters of the TyGS algorithm allow for accurate and reliable prediction of new species (cut-off = 70%) as well as subspecies (cut-off = 79%).

Also, we implemented ubcg2 pipeline^36^ consisting of 81 universal bacterial core gene set (*ala*S, *cgt*A, *dna*G, *dna*X, *eng*A, era, ffh, fmt, frr, *fts*Y, gmk, *his*S, *ile*S, i*nf*B, i*nf*C, *ksg*A, *lep*A, *leu*S, *nus*A, *nus*G, *phe*S, *phe*T, *prf*A, *rec*A, *rpl*A, *rpl*B, *rpl*C, *rpl*D, *rpl*E, *rpl*F, *rpl*I, *rpl*J, *rpl*K, *rpl*L, *rpl*M, *rpl*N, *rpl*O, *rpl*P, *rpl*Q, *rpl*R, *rpl*S, *rpl*T, *rpl*U, *rpl*V, *rpl*W, *rpl*X, *rpm*A, *rpm*C, *rpm*I, *rpo*A, *rpo*B, *rps*B, *rps*C, *rps*D, *rps*E, *rps*F, *rps*G, *rps*H, *rps*I, *rps*J, *rps*L, *rps*M, *rps*O, *rps*P, *rps*Q, *rps*R, *rps*S, *rps*T, *rsm*H, *ruv*B, *sec*A, *sec*Y, *ser*S, *smp*B, *til*S, *trm*D, *tru*B, *tsa*D, tsf, *ybe*Y, *ych*F) with default parameters to verify the robustness of the GBDP tree topology. *E. coli* ATCC 11775^T^ was used as outgroup in all the evolutionary tree inferences.

### In silico detection of secondary metabolite clusters

The whole-genome sequences of strain S1Bt23 was scanned for potential secondary metabolite biosynthesis gene clusters using antiSMASH 7.0.1 with the ‘strict’ detection parameter to obtain well-defined clusters containing all required parts^86^. The ‘Minimum Information about a Biosynthetic Gene Cluster’ (MIBiG) option in antiSMASH was also selected to access the repository of clusters of known function. Prism 4^87^ was implemented to predict the structure of the phenazine produced by strain S1Bt23. The genome of S1Bt23 was also mined for other virulence factors using “virulence factors of pathogenic bacteria” tool^39^.

### In vitro and *in planta* interactions of strain S1Bt23 with *Pythium*

Dual culture assays were used to test the antagonistic activity of strain S1Bt23 against *P. ultimum* as previously described by Tchagang et al.^28^. Briefly, a plug of *P. ultimum* (5 mm diameter) from a 48-h old culture was transferred to the center of a 90 mm Petri dish containing glucose-casamino acid-yeast (GCY; glucose 15 g/l, casamino acids 1.5 g/L, yeast extract 1.0 g/L, KH_2_PO_4_ 1.5 g/l, MgSO_4_.7H_2_O 1.0 g/L, agar 15 g/L) medium. Strain S1Bt23 was inoculated equidistantly on both sides of the culture plug and incubated at 30 °C and monitored daily for 7 days. The inhibition rate (%) was calculated using the formula: ([G_r_ − G_r+b_]/G_r_) × 100, where Gr is radial growth of *P. ultimum* alone, and G_r+b_ is the radial growth of *Pythium* towards the streaked bacterial strain S1Bt23. The assay was repeated four times with three replicates.

We, also, investigated whether strain S1Bt23 could protect potato tubers against *P. ultimum* (Py.u) using the slice inoculation technique. Yellow-fleshed potato tubers were obtained from a local grocery store in Ottawa, Canada and washed in tap water prior to surface disinfection with 70% ethanol for 5 min. The disinfected tubers were rinsed three times in autoclaved distilled water and blotted on sterile tissue paper. Equal slices were cut with a sterile knifeand treated with Py.u alone, Py.u + S1Bt23 (wild type), Py.u + S1Bt23Δ*phz*B, Py.u + S1Bt23Δ*phz*F. To test whether the bacterial strains alone could induce necrosis, tuber slices were also treated with S1Bt23, S1Bt23Δ*phz*B or S1Bt23Δ*phz*F alone without the pathogen. *Pythium* was inoculated on the slices, where required, as a 5-mm mycelial disk mat (without agar) at the point of bacterial application. The development of necrotic spots were monitored daily and diameter (d) recorded. The area of the necrotic radial lesions (A) was computed using the formula: A = π(d/2)^2^ and the statistical analysis performed with the one-way analysis of variance as implemented in https://astatsa.com/OneWay_Anova_with_TukeyHSD/. The Tukey HSD (honestly significant difference) test was used to determine the statistical significance of the data.

### pACRISPR plasmid backbone modification and cloning of the single guide RNA

The plasmids used in this study, their source (Addgene Inc) and antibiotic susceptibility are shown in Table S3. The selectable marker for the pACRISPR^40^ plasmid is based on the ampicillin resistance gene but previous antibiotic susceptibility tests on S1Bt23 revealed its resistance to this antibiotic. To overcome this bottleneck, the pACRISPR backbone was modified by replacing the ampicillin resistance gene with kanamycin resistance gene sequence using the Gibson cloning technique. The pACRISPR backbone without the amp^r^ gene was obtained by PCR using the pACRISPR-F/pACRISPR-R the primer set (Table S3). In parallel, the kanamycin resistance gene was amplified from the pGNW2^88^ plasmid (Addgene, #122086) using the pGNW2-KanR-F/pGNW2-KanR-R primer set (Table S3). Both DNA amplicons were assembled by the Gibson cloning method using NEBuilder® HiFi DNA Assembly Master Mix (New England Lab, USA). The assembled DNA sequence (pAKanCRISPR) was confirmed by Sanger sequencing using the sequencing primer: GAAACCTGTCGTGCCAGAAT. The new plasmid was then transformed into *E. coli* DH5alpha for propagation.

For the preparation and DNA cloning of the single guide RNA (sgRNA), the pAKanCRISPR vector was first digested using Bsa-HF v2 (New England Biolabs, NEB). The guide RNA (sgRNA) was then assembled and ligated into the digested pAKanCRISPR vector using a T4 DNA ligase. The successfully ligated product pAKanCRISPR-sgRNA was double-digested with *Xba*I and *Xho*I. PCR amplification was used to generate the left and right homology arm (HR) fragments using S1Bt23 WT genomic DNA as template. The homology arms were ~ 500 bp flanking the gene to be deleted. The left and right homology arms were then cloned into the digested pAKanCRISPR-sgRNA vector via Gibson assembly, resulting in the final product, pAKanCRISPR-sgRNA-HR. For cloning purposes, the high efficiency NEB® 5-alpha competent *E. coli* cells (New England Lab, USA) cells were used for plasmid propagation and preparation. Sanger sequencing using the M13/pUC-R sequencing primer set (Table S3) was used to confirm correct insertion and sequence identity.

### pAKanCRISPR–cas9 deletion of *phz*B and *phz*F genes

Electrocompetent cells of strain S1Bt23 were prepared as published by Choi et al.^89^. A 50 μL of electrocompetent cells was mixed with 100 ng of pAKanCRISPR-sgRNA-HR plasmid (containing 2–3 guides) and electroporated using Gene Pulser II Electroporator (BioRad, Canada) with parameters 1.5 kV, 200 Ω, 25 μF, in 1 mm cuvette. One ml of fresh LB medium was added immediately after electroporation and bacteria were cultured at 30 °C, 250 rpm for 1 h to promote recovery. Colonies containing the pAKanCRISPR-sgRNA-HR plasmid were selected on LB + kanamycin plates. Colony PCR was performed using specific HR1-F and M13/pUC-R primer set (Table S3) to confirm the presence of the pAKanCRISPR-sgRNA-HR plasmid. Positive colonies were selected and cultured in LB medium (10 ml) overnight.

For pCasPA transformation, electrocompetent cells were prepared as described above. The electrocompetent cells (50 μL) were mixed with pCasPA plasmid (200 ng) and electroporated as described above. One milliliter of fresh LB medium containing L-arabinose (2 mg/ml) was added immediately after electroporation to induce expression of the Cas9 nuclease and λ-Red system. Bacteria were cultured at 30 °C, 250 rpm for 2 h to promote recovery. Colonies containing pAKanCRISPR-sgRNA-HR and pCasPA were selected on LB + kanamycin + tetracycline plates. Colony PCR was performed to evaluate the successful deletion of the target genes using the following primer sets—for *phz*B gene, the set phzB-F/phzB-R and for *phz*F gene, phzF-F/phzF-R primers (Table S3) yielding the data shown in Figure S4. PCR of the franking genes were also used to validate gene deletions with the primer sets listed in Table S3. S1Bt23 (WT) was used as negative control. PCR products from potential mutant colonies were sequenced to confirm respective gene deletions. Confirmed S1Bt23 mutants were then subcultured and preserved for downstream applications.

Plasmid curing were done as follows: one colony of transformed S1Bt23 was used to inoculate 10 ml of LB broth and cultured for 16 h. The culture was serially diluted 10^–4^ fold with fresh LB medium. A 50 μL aliquot was spread on LB agar with or without 5% w/v sucrose. Three random colonies from the sucrose-containing plate were separately suspended in 35 μl LB medium. Ten microliters of bacterial culture from each dilution were streaked on LB agar plates amended with antibiotics or without (tetracycline or kanamycin). Colonies that grew on the antibiotic-free plate, but not on tetracycline or kanamycin plates were successfully cured. These colonies were then subcultured and preserved at − 80 °C in 15% glycerol.

### S1Bt23 metabolite extraction, TLC visualization and HPLC analyses

The protocol for extraction of antifungal metabolites was adapted from Mehnaz et al.^90^. Strain S1Bt23 or mutants were cultured in 200 mL of LB broth with shaking (225 rpm) for 90 h at 30 °C. Chloroform (30 ml) was added to 200 Lm of bacterial supernatant, mixed thoroughly and allowed to separate into layers. The upper (aqueous) layer was obtained and acidified with hydrochloric acid (HCl) to pH 3. Chloroform (30 mL) was added to the aqueous layer and mixed thoroughly, followed by collection of the organic phase. The previous step was repeated two more times. The organic fraction (~ 90 ml) was then washed with 30 mL ddH_2_O, followed by retention of the organic phase. The final extract was dehydrated using anhydrous sodium sulfate (6 g), decanted and allowed to evaporate completely. The extract residues were re-dissolved in 500 μL methanol and transferred into 1.5-ml Eppendorf tubes for storage at 20 °C.

S1Bt23 extracts (20 μL) were applied on a 20 cm × 10 cm glass silica60 gel plate (Sigma, Canada). Synthetic phenazine-1-carboxylic acid (5 mg/mL) of 95% purity (Sigma) was used as positive control, while methanol was used as negative control. The silica plate was placed into a glass TLC chamber containing chloroform:acetic acid (49:1, v/v) as solvent (mobile phase). After about 2 h of sample migration, the silica plate was dried in a fume hood for 30 min. at room temperature in the dark, followed by imaging with a UV transilluminator.

Phenazine qualification and quantification analyses were performed on an Agilent HPLC system (1100 Series, with quaternary pump and autosampler) equipped with a variable wavelength detector (VWD, G1314A, Agilent Series 1100) and a diode array detector (DAD, G1315D, Agilent 1260 Infinity Series). An Atlantis T3 column, 2.1 mm × 100 mm, 3 μm pore size (Waters, Milford, MA) was used with correspondent guard column (column T: 30 °C). Two solvents were used in the mobile phase with the following gradient: starting with 15% acetonitrile/85% formic acid (0.1% w/v in MilliQ water), acetonitrile ramped up to 80% by min 20, reached to 100% at min 22, decreased to 15% by min 24, and equilibrated the column with that composition until min 30. Phenazine peaks were identified by comparison of the retention time and UV spectra with the standard compound (95% purity; cat# 2538-68-3; Sigma), and the concentration was calculated using a standard curve (at 248 nm) generated from the standard.

### Morphology and phenotypic characterization

Cell morphology of strain S1Bt23 was determined by scanning (SEM) and transmission (TEM) electron microscopy as described previously by Tambong et al.^21^. Gram status was determined using the 3% KOH test [Ryu 1940]. The optimum growth temperature of the strain was evaluated in the range of 5 °C to 41 °C. Carbon utilization and enzymatic tests of strain S1Bt23 were performed, in parallel, with the three other subspecies of *P. chlororaphis* in triplicate, using Biolog plates PM1 & PM2A (Hayward, CA) and API 20NE (BioMérieux, Canada), respectively, according to the manufacturers' instructions. pH and salt tolerance were evaluated in the Biolog Gen III system. Polar lipids of strain S1Bt23 were determined by the Identification Service of the DSMZ (Braunschweig, Germany). pH and salt tolerance were determined using the GEN III system (Biolog, USA).

## Supplementary Information

Supplementary Information.

## Data Availability

Sequence data that support the findings of this study have been deposited in NCBI GenBank Nucleotide Archive with accession numbers: CP139026 (whole genome) under bioproject number PRJNA1039847, 16S rRNA (MH463705), rpoD (MH494124), gyrB (MH544559) and rpoB (MH487805).

## Plant material declaration

All the plant experiments/protocols were performed with relevant institutional, national, and international guidelines and legislation.
