# Supplementary material for: Phenazines are involved in the antagonism of a novel subspecies of Pseudomonas chlororaphis strain S1Bt23 against Pythium ultimum
Source: Sci Rep. 2024 Sep 3;14:20517. doi: 10.1038/s41598-024-71418-y (PMC11372166; doi:10.1038/s41598-024-71418-y)
Supplement: Supplementary file 1 — Supplementary Information. [file 41598_2024_71418_MOESM1_ESM.pdf]

Table S1. GenBank accession numbers of the 135 whole genome sequences of *Pseudomonas chlororaphis* analyzed in this study.

Organism Name	Strain	Level	Size(Mb)	GC%	NCBI accession numbers	Number of replicons /scaffolds	CDS
Pseudomonas chlororaphis	ATCC 336631	Complete	7.10935	62.9	CP118152	1	6286
Pseudomonas chlororaphis	ATCC 336632	Complete	7.10882	62.9	CP118140	1	6286
Pseudomonas chlororaphis	DSM 21509	Complete	7.06497	62.6	CP118150	1	6256
Pseudomonas chlororaphis	ATCC 17809	Complete	7.0209	62.4	CP118142	1	6223
Pseudomonas chlororaphis	B25	Complete	7.01659	62.0	CP027753	1	6038
Pseudomonas chlororaphis	PCLSL01	Complete	6.98922	62.7	CP054865	1	6122
Pseudomonas chlororaphis	NCCB 60038	Complete	6.97935	62.7	CP118136	1	6213
Pseudomonas chlororaphis	NCCB 60037	Complete	6.97728	62.7	CP118149	1	6212
Pseudomonas chlororaphis	NCCB 880622	Complete	6.92323	62.8	CP118148	1	6146
Pseudomonas chlororaphis	ATCC 174181	Complete	6.88327	62.8	CP118144	1	6085
Pseudomonas chlororaphis	ATCC 174182	Complete	6.88164	62.8	CP118143	1	6085
Pseudomonas chlororaphis	L5734	Complete	6.88047	62.8	CP083442	1	5960
Pseudomonas chlororaphis	189	Complete	6.83778	62.7	CP014867	1	5882
Pseudomonas chlororaphis	Lzh-T5	Complete	6.82669	63.1	CP025309	1	5960
Pseudomonas chlororaphis	ATCC 17814	Complete	6.80791	63.0	CP118141	1	6065
Pseudomonas chlororaphis	ATCC 17414	Complete	6.80717	63.0	CP118154	1	6067
Pseudomonas chlororaphis	X8	Complete	6.77729	63.0	CP077678	1	6022
Pseudomonas chlororaphis	ATCC 15926	Complete	6.76392	62.9	CP118156	1	5999
Pseudomonas chlororaphis	NCCB 820532	Complete	6.76216	62.9	CP118135	1	5989
Pseudomonas chlororaphis	ATCC 17417	Complete	6.74654	62.9	CP118145	1	5963
Pseudomonas chlororaphis	TAMOak81	Complete	6.71136	63.0	CP027713	1	5949
Pseudomonas chlororaphis	ATCC 17415	Complete	6.67088	63.0	CP027714	1	5862
Pseudomonas chlororaphis	ATCC 174152	Complete	6.6645	63.0	CP118146	1	5884
Pseudomonas chlororaphis	ATCC 174151	Complete	6.66416	63.0	CP118147	1	5885
Pseudomonas chlororaphis	PCLRT02	Complete	6.66345	63.1	CP054867	1	5850
Pseudomonas chlororaphis	PCL1606	Complete	6.6629	64.0	CP011110	2	5926
Pseudomonas chlororaphis	Pb-St2	Complete	6.6138	63.2	CP027716	1	5793
Pseudomonas chlororaphis	PcR3-3(2)	Complete	7.38216	62.5	CP128834	1	6531
Pseudomonas chlororaphis	PCLAR04	Complete	7.30565	62.6	CP054868	1	6446
Pseudomonas chlororaphis	NCCB 47033	Complete	7.22153	62.4	CP118137	1	6376
Pseudomonas chlororaphis	DSM 295781	Complete	7.21695	62.5	CP118139	1	6376
Pseudomonas chlororaphis	DSM 295782	Complete	7.21657	62.5	CP118138	1	6375
Pseudomonas chlororaphis	ATCC 17411	Complete	7.21242	62.5	CP118155	1	6371
Pseudomonas chlororaphis	NCTC7357	Complete	7.20993	62.5	LR134334.1	1	6351
Pseudomonas chlororaphis	R47	Complete	7.1973	62.6	CP019399	1	6321
Pseudomonas chlororaphis	ATCC 9446	Complete	6.63779	63.0	CP118151	1	5927
Pseudomonas chlororaphis	PCLAR05	Contig	6.69917	63.1	JABVYF01	8	5851
Pseudomonas chlororaphis	WS 5014	Contig	6.78762	62.8	JAAQZE01	31	5911
Pseudomonas chlororaphis	48B8	Scaffold	6.65216	63.0	MOAO01	97	5900
Pseudomonas chlororaphis	14D6	Scaffold	7.0126	62.4	MOAL01	18	6236
Pseudomonas chlororaphis	FW305-25	Contig	6.97211	62.7	FMCZ01	22	6250
Pseudomonas chlororaphis	FER02	Contig	7.10175	61.4	JAHTLD01	30	6075
Pseudomonas chlororaphis	L19	Scaffold	6.89812	62.8	LNTS01	4	6088
Pseudomonas chlororaphis	LBs-160603	Scaffold	6.80198	62.9	QJRW01	25	6025
Pseudomonas chlororaphis	PCL1601	Contig	6.75544	63.6	MSCT01	25	5911
Pseudomonas chlororaphis	VE267-6B	Contig	6.8227	62.0	JAMYBM01	56	6064
Pseudomonas chlororaphis	S265-5	Contig	6.82168	62.0	JAMYBP01	55	6064
Pseudomonas chlororaphis	JV497	Contig	6.87565	62.9	VWPC01	32	6108
Pseudomonas chlororaphis	VA158-3	Contig	6.91493	62.8	JAMYBL01	52	6210
Pseudomonas chlororaphis	S265-4	Contig	6.89534	62.9	JAMYBO01	64	6144
Pseudomonas chlororaphis	S265-2	Contig	6.89514	62.9	JAMYBN01	63	6142
Pseudomonas chlororaphis	HAMBI_1977	Scaffold	6.62182	63.1	QLKZ01	38	5864
Pseudomonas chlororaphis	PCLRT01	Contig	6.90122	62.8	JABVYE01	92	6034
Pseudomonas chlororaphis	EA105	Scaffold	6.59558	59.2	JSFK01	74	5731
Pseudomonas chlororaphis	388_19	Scaffold	7.15365	63.7	JAHRBT01	99	6429
Pseudomonas chlororaphis	JV395B	Contig	6.77101	63.1	VWPB01	184	6054
Pseudomonas chlororaphis	PCLRT03	Contig	7.31369	62.4	JABVYD01	394	6391
Pseudomonas chlororaphis	PCLAR01	Contig	7.57037	62.5	JABVYI01	398	6709
Pseudomonas chlororaphis	PCLAR02	Contig	7.74082	62.4	JABVYH01	628	6879
Pseudomonas chlororaphis	PCLAR03	Contig	7.42581	62.4	JABVYG01	1081	6762
Pseudomonas chlororaphis	14B11	Scaffold	6.69627	63.0	MOAN01	109	6131
Pseudomonas chlororaphis	48G9	Scaffold	6.652	63.0	MOBW01	93	6121
Pseudomonas chlororaphis	ATCC 9446	complete	6.81828	63.0	LT852426	1	6070
Pseudomonas chlororaphis	LMG 21630	complete	7.12059	62.9	LT629747	1	6275
Pseudomonas chlororaphis	DSM 21509	complete	7.0652	62.6	LT629761	1	6221
Pseudomonas chlororaphis	ATCC 13985	complete	7.04762	62.7	LT629738	1	6243
Pseudomonas chlororaphis	PCLRT04	complete	6.95766	62.6	CP054866	1	6074
Pseudomonas chlororaphis	DSM 50083	Scaffold	6.76659	63.0	JADCLH01	32	6019
Pseudomonas chlororaphis	qlu-1	Complete	6.82768	63.1	CP061079	1	5969
Pseudomonas chlororaphis	NCIB 10068_1	Contig	6.71876	62.9	JALJWZ01	1	5994
Pseudomonas chlororaphis	NCIB 10068_2	Contig	6.71875	62.9	JALJWS01	1	5998
Pseudomonas chlororaphis	NCTC10686	Contig	7.03117	62.7	UGUQ01	3	6254
Pseudomonas chlororaphis	ATCC 17811	Complete	7.18911	62.4	CP118153	1	6385
Pseudomonas chlororaphis	PA23	Complete	7.12217	62.6	CP008696	1	6322
Pseudomonas chlororaphis	UFB2	Complete	6.36026	62.0	CP011020	1	5476
Pseudomonas chlororaphis	HT66	Contig	7.29862	62.6	ATBG01	50	6468
Pseudomonas chlororaphis	O6	complete	6.98025	62.9	CM001490	1	12421
Pseudomonas chlororaphis	YL-1	Scaffold	6.80098	63.1	AWWJ01	82	6111

Table S1 cont'd

Pseudomonas chlororaphis subsp. aurantiaca	DSM 19603	Complete	7.10978	62.9	CP027746	1	6263
Pseudomonas chlororaphis subsp. aurantiaca	SMMP3	Scaffold	6.85701	62.9	JAEEFW01	25	6012
Pseudomonas chlororaphis subsp. aurantiaca	PB-St2	Scaffold	6.59092	63.2	AYUD01	23	5795
Pseudomonas chlororaphis subsp. aurantiaca	Tre132	Complete	6.9617	62.8	AP020336	1	6519
Pseudomonas chlororaphis subsp. aurantiaca	K27	Complete	6.8677	62.8	CP027745	1	5913
Pseudomonas chlororaphis subsp. aurantiaca	M71	Complete	6.807	62.9	CP027744	1	6004
Pseudomonas chlororaphis subsp. aurantiaca	M12	Complete	6.73098	63.0	CP027715	1	5939
Pseudomonas chlororaphis subsp. aurantiaca	JD37	Complete	6.70206	62.8	CP009290	1	5914
Pseudomonas chlororaphis subsp. aurantiaca	ARS 38	Complete	6.61505	63.2	CP045221	1	5797
Pseudomonas chlororaphis subsp. aurantiaca	B-162	complete	7.10987	62.9	CP050510	1	6270
Pseudomonas chlororaphis subsp. aurantiaca	StFRB508	Complete	6.99793	62.8	AP014623	1	6201
Pseudomonas chlororaphis subsp. aurantiaca	464	Complete	6.96445	62.9	CP027742	1	6131
Pseudomonas chlororaphis subsp. aurantiaca	449	Complete	6.96207	62.9	CP027741	1	6126
Pseudomonas chlororaphis subsp. aurantiaca	YS05	Complete	6.95303	62.5	CP059162	1	6192
Pseudomonas chlororaphis subsp. aurantiaca	Q16	Complete	6.92855	62.8	CP027718	1	6040
Pseudomonas chlororaphis subsp. aurantiaca	CW2	Complete	6.9252	62.8	CP027743	1	6103
Pseudomonas chlororaphis subsp. aurantiaca	YS21	Complete	6.91594	62.8	CP068390	1	6087
Pseudomonas chlororaphis subsp. aurantiaca	zm-1	Complete	6.86133	63.0	CP048051	1	6090
Pseudomonas chlororaphis subsp. aurantiaca	PCM 2210	Complete	6.85448	63.0	CP027717	1	6030
Pseudomonas chlororaphis subsp. aureofaciens	DSM 6698	Complete	7.03785	62.7	CP027720	1	6241
Pseudomonas chlororaphis subsp. aureofaciens	CD	Contig	6.80214	63.0	LHVB01	32	6025
Pseudomonas chlororaphis subsp. aureofaciens	ChPhzS23	Complete	6.75609	63.0	CP027748	1	5966
Pseudomonas chlororaphis subsp. aureofaciens	P2	Complete	7.20306	62.8	CP027719	1	6377
Pseudomonas chlororaphis subsp. aureofaciens	ChPhzTR39	Complete	7.0461	62.7	CP027749	1	5982
Pseudomonas chlororaphis subsp. aureofaciens	ChPhzTR38	Complete	6.94772	62.8	CP027752	1	6105
Pseudomonas chlororaphis subsp. aureofaciens	ChPhzTR36	Complete	6.93408	62.7	CP027721	1	6041
Pseudomonas chlororaphis subsp. aureofaciens	ChPhzS24	Complete	6.88955	62.9	CP027750	1	6036
Pseudomonas chlororaphis subsp. aureofaciens	ChPhzTR18	Complete	6.8732	62.9	CP027751	1	6107
Pseudomonas chlororaphis subsp. aureofaciens	66	Complete	6.79728	63.0	CP027747	1	6015
Pseudomonas chlororaphis subsp. aureofaciens	SPS-41	Complete	6.7579	63.1	CP066546	1	5929
Pseudomonas chlororaphis subsp. aureofaciens	C50	Complete	6.74127	63.0	CP027722	1	5935
Pseudomonas chlororaphis subsp. aureofaciens	LMG 1245	Contig	7.02285	62.7	LHVA01	45	6260
Pseudomonas chlororaphis subsp. aureofaciens	CCUG 712	Contig	7.00595	62.7	VZPK01	100	6272
Pseudomonas chlororaphis subsp. aureofaciens	30-84	complete	6.66632	62.9	CM001559	1	11707
Pseudomonas chlororaphis subsp. aureofaciens	NBRC 3521	Contig	6.97334	62.8	BBQB01	59	6246
Pseudomonas chlororaphis subsp. chlororaphis	DSM 50083	Complete	6.80819	63.0	CP027712	1	6045
Pseudomonas chlororaphis subsp. chlororaphis	LMG 5004	Contig	6.79247	63.0	LHVC01	15	6058
Pseudomonas chlororaphis subsp. chlororaphis	DSM 50083	Contig	6.7856	63.0	VFIN01	25	6067
Pseudomonas chlororaphis subsp. chlororaphis	DSM 50083T	Contig	6.78377	63.0	UYXS01	25	6059
Pseudomonas chlororaphis subsp. chlororaphis	ATCC 9446	Contig	6.78303	63.0	NBAT01	35	6065
Pseudomonas chlororaphis subsp. chlororaphis	GP72	Contig	6.62988	63.1	AHAY01	347	5977
Pseudomonas chlororaphis subsp. chlororaphis	NBRC 3904	Contig	6.77165	63.0	BCZX01	36	6072
Pseudomonas chlororaphis subsp. piscium	DSM 21509	Complete	7.06515	62.6	CP027707	1	6245
Pseudomonas chlororaphis subsp. piscium	PCL1391	Contig	6.85556	62.8	LFUT01	17	6013
Pseudomonas chlororaphis subsp. piscium	PS5	Complete	7.10791	62.7	CP097271	1	5377
Pseudomonas chlororaphis subsp. piscium	SLPH10	Complete	7.22764	62.5	CP027710	1	6402
Pseudomonas chlororaphis subsp. piscium	ATCC 17809	Complete	7.21889	62.4	CP027709	1	6358
Pseudomonas chlororaphis subsp. piscium	ATCC 17411	Complete	7.2124	62.5	CP027708	1	6340
Pseudomonas chlororaphis subsp. piscium	ChPhzS140	Complete	7.07432	62.6	CP027740	1	6238
Pseudomonas chlororaphis subsp. piscium	DTR133	Complete	7.06462	62.7	CP027735	1	6171
Pseudomonas chlororaphis subsp. piscium	ToZa7	Complete	7.0156	62.6	CP027739	1	6174
Pseudomonas chlororaphis subsp. piscium	ChPhzS135	Complete	6.94002	62.8	CP027738	1	6056
Pseudomonas chlororaphis subsp. piscium	PCL1607	Complete	6.91364	62.8	CP027737	1	6079
Pseudomonas chlororaphis subsp. piscium	ChPhzTR44	Complete	6.87822	62.8	CP027711	1	6046
Pseudomonas chlororaphis subsp. piscium	PCL1391	Complete	6.87062	62.8	CP027736	1	6004
Pseudomonas chlororaphis subsp. piscium	ZJU60	Complete	6.818	62.8	CP027656	1	6021
Pseudomonas chlororaphis subsp. piscium	DSM 21509	Contig	7.04461	62.7	LHUZ01	36	6251

Table S2. Cellular fatty acid composition (%) of *Pseudomonas chlororaphis* subsp. *phenazini* subsp. nov. S1Bt23^T and other *P. chlororaphis* subspecies. Strains: 1, S1Bt23^T (=CFBP 9180^T); 2, subspecies *chlororaphis* LMG 5004^T; 3, subsp. *aurantiaca* LMG 21630^T; 4, subsp. *aureofaciens* LMG 1245^T; and 5, subsp. *piscium* DSM 21509^T.

**Fatty acid**	**1**	**2**	**3**	**4**	**5**
$C_{10:0}$ 3-OH	6.4	4.4	7.3	5.0	7.6
$C_{12:0}$	1.6	1.6	1.7	1.6	1.8
$C_{12:0}$ 2OH	5.5	5.31	5.3	5.2	6.18
$C_{12:0}$ 3OH	6.2	4.3	6.4	4.9	6.0
$C_{16:0}$	25.9	29.6	28.1	29.4	25.9
$C_{17:0}$ cyclo	3.0	1.1	4.7	3.9	0.72
$C_{16:1}$ w7c/$C_{16:1}$ w6c†	30.0	36.9	28.1	33.9	31.1
$C_{18:1}$ w7c /$C_{18:1}$ w6c$^{\#}$	14.5	15.2	12.1	12.7	13.6

†summed feature 3; $^{\#}$ summed feature 8.

Table S3. Bacterial strains, plasmids and primers used in this study

strains/plasmids/primers	Characteristics or Sequence 5' ->3' orientation	Description	Source
**Bacterial strains:**			
Pseudomonas chlororaphis:			
S1Bt23	wild type, Phz+, Car^r, Strept^r, Kan^s, tet^s	wild type, potent antagonist of *Pythium ultimum*	This study
S1Bt23ΔphzB	mutant, ΔphzB, Phz-	S1Bt23 derivative with *phz* B deleted	This study
S1Bt23ΔphzF	mutant, ΔphzF, Phz-	S1Bt23 derivative with *phz* F deleted	This study
Escherichia coli :	*E. coli* DH5alpha	Carrier of the plasmids	Addgene
**Plasmids:**			
pCasPA	Tet^r	Expression of Cas9 nuclease and λ-Red system	Addgene # 113347*
pACRISPR	car^r, amp^r, Kan^s	backbone for sgRNA expression kanamycin susceptible	Addgene # 113348*
pAKanCRISPR	pACRISPR plus Kan^r, amp^s	backbone for sgRNA expression with kanamycin resistance	This study
pGNW2	Kan^r	source of kanamycin resistant gene	AddGene #122086*
**Oligonucleotides:**			
pGNW2-KanR-F	aggtggcacttttcggggaaatgtgATGGCGATAGCTAGACTGGG	To amplify the Kanamycin resistance (KanR) gene from pGNW2 plasmid	This study
pGNW2-KanR-R	tatatgagtaaacttggtctgacagTCAGAAGAACTCGTCAAGAAG		
pACRISPR-F	CTGTCAGACCAAGTTTACTC	To amplify the pACRISPR backbone without the ampicillin resistance (AmpR) gene	This study
pACRISPR-R	CACATTTCCCCGAAAAGTGC		
phz B CRISPR knockout:			
phzB guide 1-F	GTGGTGGGTCGAGTGCGATGGCCG	phzB guide RNA 1 (sgRNA1)	This study
phzB guide 1-R	AAACCGGCCATCGCACTCGACCCA		
phzB guide 2-F	GTGGGGAGGTTGTTGTATGTATGG	phzB guide RNA 2 (sgRNA2)	This study
phzB guide 2-R	AAACCCATACATACAACAACCTCC		
phzB HR1-F	ttttgagatctgtccatacccatggTCTAGACCCAACTAAGGAGGATGCTG	To amplify the left homology arm sequence of *phz* B gene	This study
phzB HR1-R	CTGCTGGAACGCTTCACAATACCTTCACGCTTGATTCTTGGAA		
phzB HR2-F	TTCCAAGAATCAAGCGTGAAGGTATTGTGAAGCGTTCCAGCA	To amplify the right homology arm sequence of *phz* B gene	This study
phzB HR2-R	tctgaatggcgggagtatgaaaagtCTCGAGTGAGTTGTTCCTGGCTTGC		
phz F CRISPR knockout:			
phzF guide 1-F	gtggTATCGGCTGGTCCATGCTGG	phzF guide RNA 1	This study
phzF guide 1-R	AAACCCAGCATGGACCAGCCGATA		
phzF guide 2-F	gtggGAAGGGCAGTTCGTTGACCG	phzF guide RNA 2	This study
phzF guide 2-R	AAACCGGTCAACGAACTGCCCTTC		
phzF guide 3-F	gtggCTTCGCCGGGCACCCGTTGC	phzF guide RNA 3	This study
phzF guide 3-R	AAACGCAACGGGTGCCCGGCGAAG		
phzF HR1-F	tttgagatctgtccatacccatggtCTAGAGCAAGGTCGGCCCGGACA	To amplify the left homology arm sequence of *phz* F gene	This study
phzF HR1-R	cggtctggctcatGACGACAAACTCCAGTCAAAAGGAGG		
phzF HR2-F	gagtttgtcgtcATGAGCCAGACCGCAGCC	To amplify the right homology arm sequence of *phz* F gene	This study
phzF HR2-R	ctgaatggcgggagtatgaaaagtcTCGAGAACGACAGCGCACTGGCC		
Confirmation of correct insertion:			
M13/pUC-R	agcggataacaatttcacacagg	Sequencing primer to confirm correct insertion of guide RNA and homology repair sequences into pACRISPR vector	This study
Confirmation of knockout:			
phzB-F	GTAATCAGTGGCAAAGCCAAG	To amplify a portion of *phz* B gene (position 181 -> 492bp)	This study
phzB-R	TTAAGTTGGAATGCCTTCGCGC		
phzF-F	ATGCACCATTACGTCATCATC	To amplify *phz* F gene (position 1 -> 837bp)	This study
phzF-R	TCATAGAACGATGGTCCCCCG		
phzA-F	ATGCCTGCTTCGCTTTCCCC	To amplify the sequences flanking *phz* B gene (phzA -> phzC)	This study
phzC-R	TCAAAAGGAGGCAAGGGTTG		
phzE-F	ATGAGCCAGACCGCAGCCC	To amplify the sequences flanking *phz* F gene (phzE -> phzG)	This study
phzG-R	CTATGGCTGTAACCGGCGATG		

Car^r, Carbenicillin resistant, Car^s , Carbenicillin susceptible; Kan^r, kanamycin resistant, Kan^s, kanamycin susceptible; Tet^r, tetacycline resistant, Strept^r, streptomycin resistant. * Addgene # 113347 and 113347 donated Chen et al.[30]; and AddGene #122086 donated by Wirth *et al* .[73].

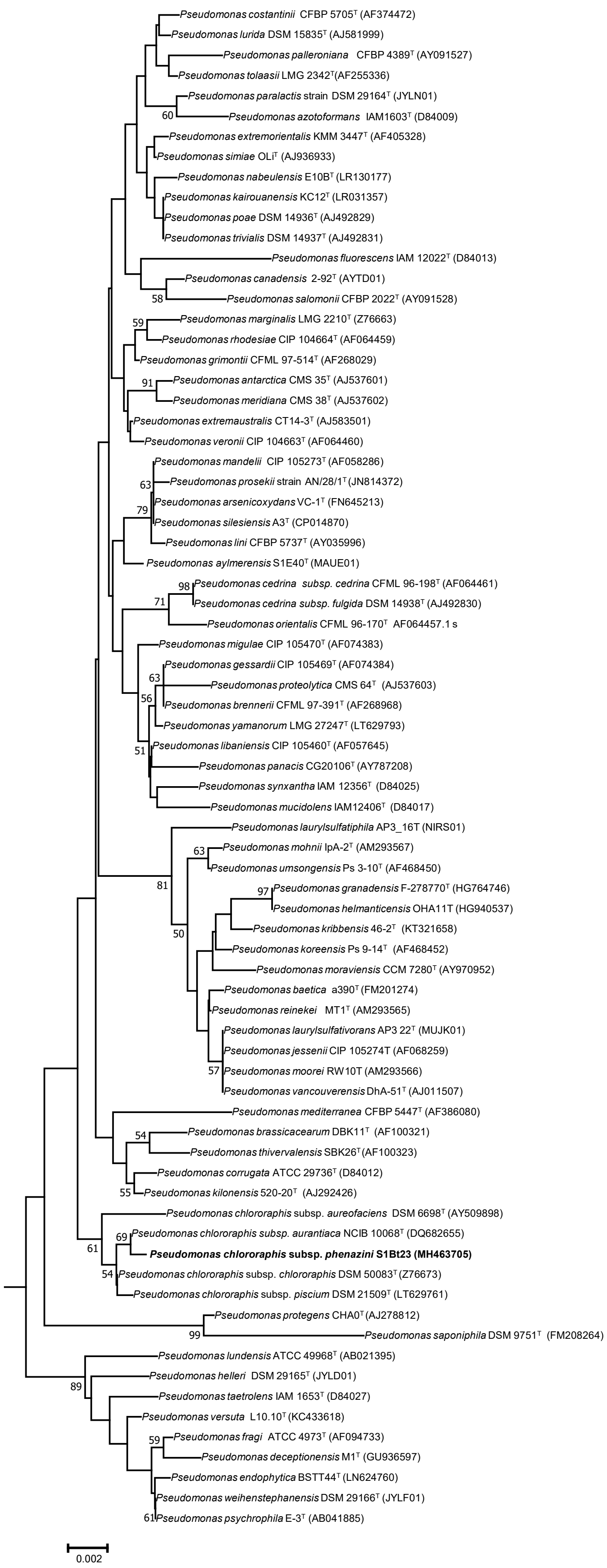

Figure S1. Maximum likelihood evolutionary tree of the 16S rRNA gene sequence showing that strain S1Bt23 clustered with members of the species *Pseudomonas chlororaphis* using Jukes and Cantor substitution model. The percentage of replicate trees in which the associated taxa clustered together in the bootstrap test (1000 replicates) are shown next to the branches. Tree rooted with *Escherichia coli* ATCC 11775^T (X80725).

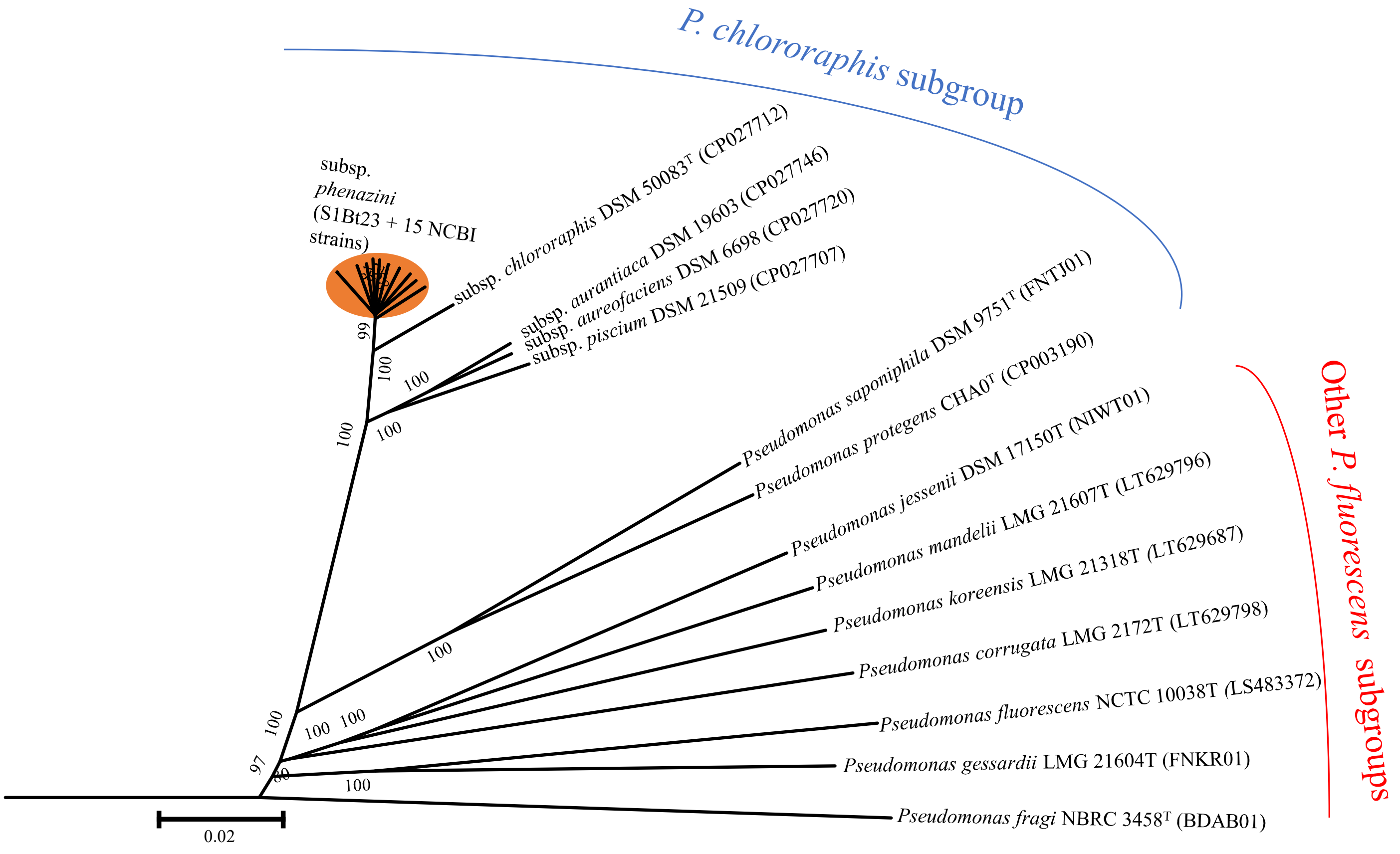

Figure S2. Genome-based TYGS-generated tree showing distinct evolutionary clustering of *Pseudomonas chlororaphis* subsp. *phenazini* strains (S1Bt23 and 14 classified only as *P. chlororaphis* and 1 as subsp. *aureofaciens* 30-84 in NCBI) relative to other members of the *P. chlororaphis* subgroup and representatives of the other *P. fluorescens* subgroups. The 14 P. chlororaphis strains are ATCC 15926, NCCB 820532, ATCC 17417, NCIB 10068_1, NCIB 10068_2, ATCC 174182, 48B8, 48G9, 14B11, ATCC 17415, ATCC 174152, ATCC 174151, HAMI_1977 and isolate 182. The TYGS algorithm classified S1Bt23 and 15 NCBI strains (orange oval) as novel subspecies based on the cut-off for subspecies of 79.0%. Branch lengths are scaled according to the GBDP distance formula d_5. Numbers above branches are GBDP pseudo-bootstrap support values > 60 % from 100 replicates. The tree was rooted at the midpoint.

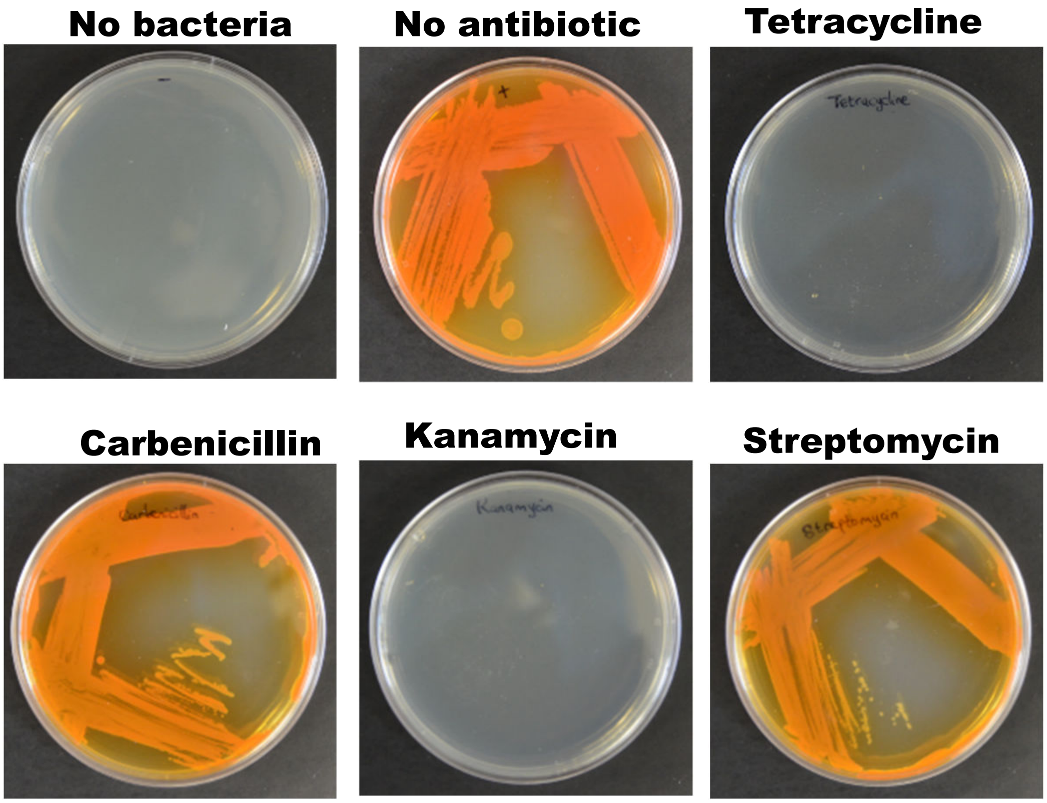

Figure S3. Antibiotic screening of strain S1Bt23 revealed resistance to carbenicillin (150 µg/ml) and streptomycin (100 µg/ml) but sensitive to tetracycline (100 µg/ml) and kanamycin (50 µg/ml). Kanamycin and tetracycline antibiotics were selected as the marker for the selection of the mutants. Plates were incubated at 30°C for 72 hrs.

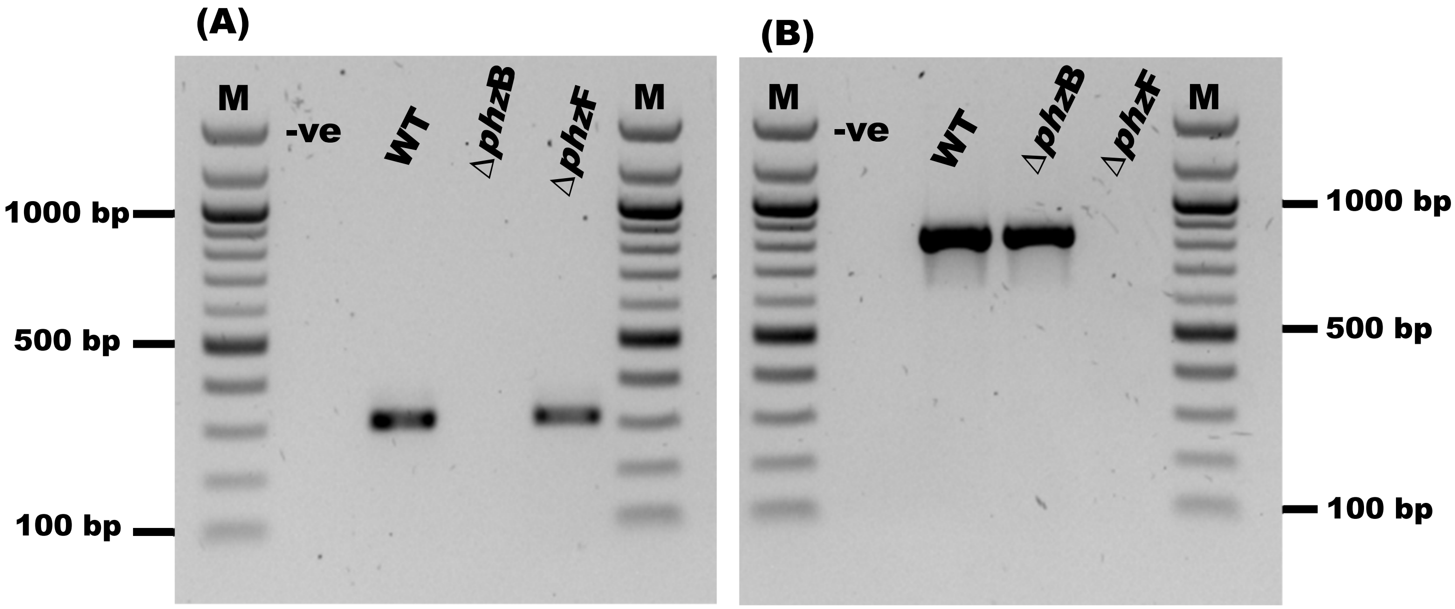

Figure S4. Confirmation of CRISPR-cas9 knockout of the *phz*B (**A**) or *phz*F (**B**) gene in strain S1Bt23 by PCR amplification using specific primer sets PhzB-F/PhzB-R or PhzF-F/PhzF-R for *phz*B and *phz*F, respectively. Note the presence of *phz*F and the absence of *phz*B amplicons in *phz*B mutant and vice versa. M, Quick-Load® Purple 100 bp DNA ladder; -ve, negative; and WT, S1Bt23 wild type.

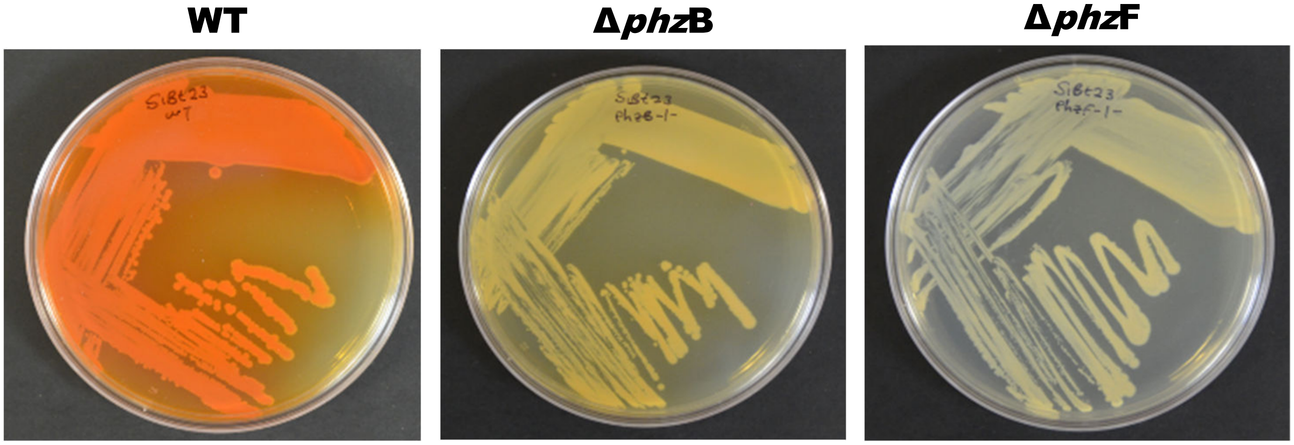

Figure S5. Strains S1Bt23Δ*phz*B and S1Bt23Δ*phz*F mutants on Luria and Bertini (LB) agar plates showing loss of characteristic orange pigmentation compared to the wild type (WT) of *Pseudomonas chlororaphis* subsp. *phenazini* strain S1Bt23. Plates were incubated at 30°C for 72 hrs.

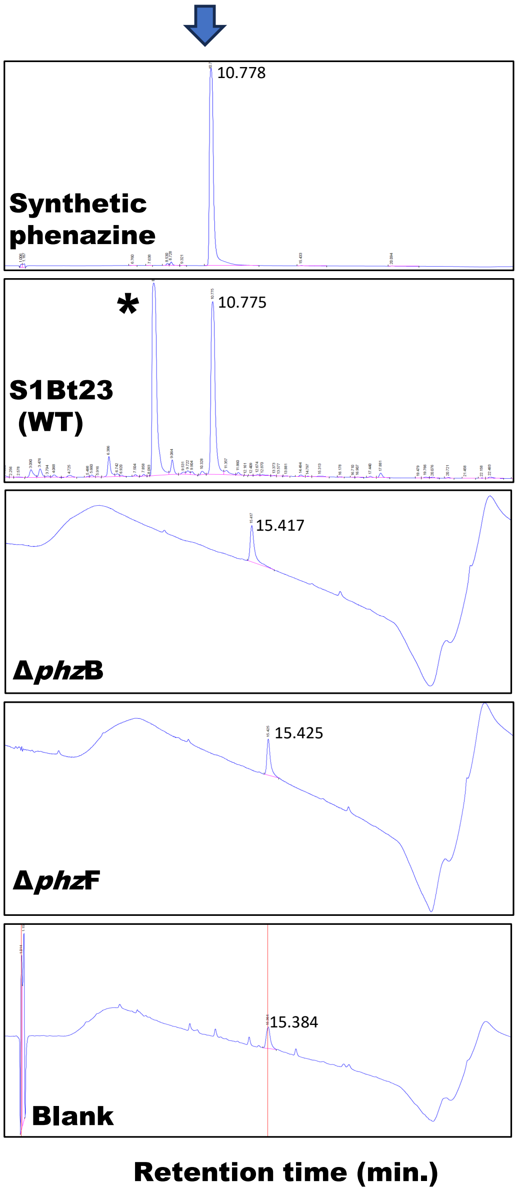

Fig. S6. High performance liquid chromatography profiles with phenazine peak detected at 10.78 (blue arrow) retention time for only the synthetic phenazine (positive control) and S1Bt23 (wild type) but not for the S1Bt23Δ*phz*B and S1Bt23Δ*phz*F mutants. * could indicate the production of either 2-hydroxphenazine-1-carboxylic acid or 2-hydroxphenazine.

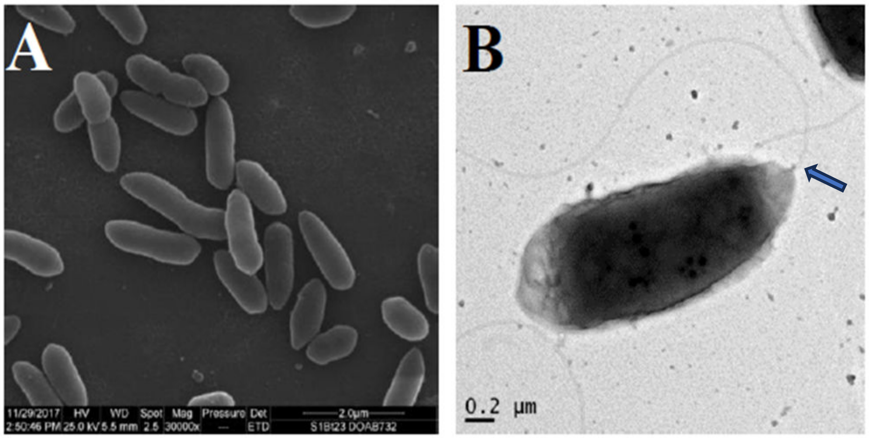

Figure S7. Cell morphology of *Pseudomonas chlororaphis* subsp. *phenazini* S1Bt23: (A) scanning electron microscopy showing the typical rod shape and (B) transmission electron microscopy with a single flagellum (arrow).

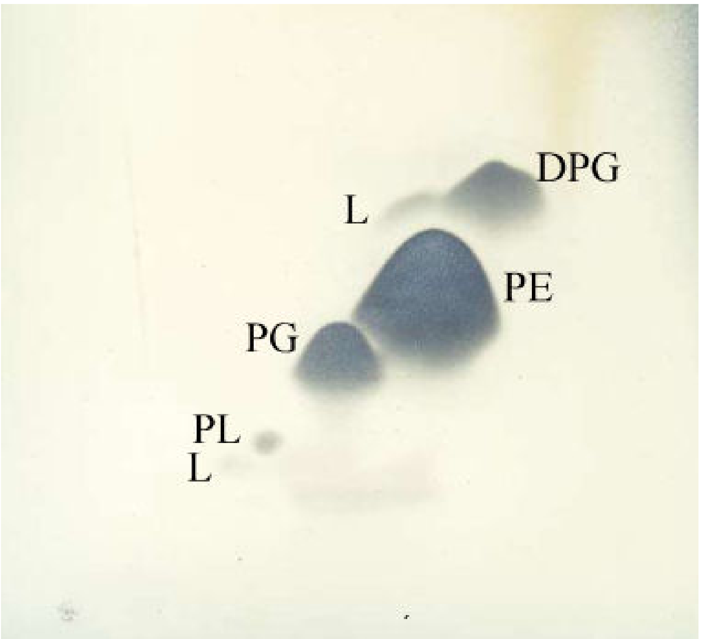

Figure S8. Polar lipids detected in the cells of *Pseudomonas chlororaphis* subsp. *phenazini* S1Bt23: PE, phosphatidylethanolamine; DPG, diphosphatidylglycerol; PG, phosphatidylglycerol; PL, phospholipids and L, lipids.
